# Pilot of a team-based quality improvement strategy to improve cardiovascular risk factors care in community mental health centers

**DOI:** 10.3389/fpsyt.2025.1446985

**Published:** 2025-01-31

**Authors:** Karly A. Murphy, Joseph Gennusa, Arlene T. Dalcin, Courtney Cook, Stacy Goldsholl, Tyler Fink, Gail L. Daumit, Nae-Yuh Wang, David Thompson, Emma E. McGinty

**Affiliations:** ^1^ Division of General Internal Medicine, Department of Medicine, University of California, San Francisco, San Francisco, CA, United States; ^2^ Multiethnic Health Equity Research Center, University of California, San Francisco, San Francisco, CA, United States; ^3^ Division of General Internal Medicine, Department of Medicine, Johns Hopkins University School of Medicine, Baltimore, MD, United States; ^4^ Welch Center for Prevention, Epidemiology and Clinical Research, Johns Hopkins University, Baltimore, MD, United States; ^5^ Department of Psychiatry and Behavioral Sciences, Johns Hopkins University School of Medicine, Baltimore, MD, United States; ^6^ Department of Epidemiology, Johns Hopkins Bloomberg School of Public Health, Baltimore, MD, United States; ^7^ Department of Health Policy and Management, Johns Hopkins Bloomberg School of Public Health, Baltimore, MD, United States; ^8^ Department of Mental Health, Johns Hopkins Bloomberg School of Public Health, Baltimore, MD, United States; ^9^ Department of Biostatistics, Johns Hopkins Bloomberg School of Public Health, Baltimore, MD, United States; ^10^ Institute for Clinical and Translational Research, Johns Hopkins University, Baltimore, MD, United States; ^11^ Department of Anesthesiology and Critical Care Medicine, Johns Hopkins University School of Medicine, Baltimore, MD, United States; ^12^ Armstrong Institute for Patient Safety and Quality, Johns Hopkins University School of Medicine, Baltimore, MD, United States; ^13^ Division of Health Policy and Economics, Department of Population Health Sciences, Weill Cornell Medical College, New York, NY, United States

**Keywords:** serious mental illness, cardiovascular risk, quality improvement, care coordination, primary care, team-based care, pilot projects, evidence-based medicine

## Abstract

**Introduction:**

Populations with serious mental illness are less likely to receive evidence-based care for cardiovascular disease (CVD) risk factors. We sought to characterize the implementation of an adapted team-based quality improvement strategy to improve mental health providers’ delivery of evidence-based CVD risk factor care.

**Methods:**

In a 12-month, single arm pre/post pilot study in four behavioral health homes embedded within psychiatric rehabilitation programs, sites implemented an adapted Comprehensive Unit Safety Program (CUSP). Primary measures examined changes in organizational quality improvement culture and provider self-efficacy for CVD risk factor care. Secondary measures examined changes in acceptability, appropriateness, and feasibility of CUSP and receipt of guideline-concordant care for hypertension, dyslipidemia, and diabetes.

**Results:**

Provider self-efficacy to coordinate care for hypertension and diabetes improved, but organizational quality improvement culture did not change. Acceptability, appropriateness, and feasibility were rated highly but did not change pre/post CUSP. The percentage who reached goals per national guidelines improved for those with dyslipidemia but not for those with hypertension or diabetes. CUSP teams implemented processes to build staff capacity, standardize communication, elicit feedback, and deliver education on coordination for CVD risk factors.

**Conclusion:**

This pilot study showed no effects of CUSP on organizational quality improvement culture or provider self-efficacy, the mechanisms by which CUSP is expected to improve care processes. Long term investments are needed to support organizational quality improvement work and providers’ efficacy to delivery - evidence-based CVD risk factor care delivery.

**Clinical Trial Registration:**

http://www.ClinicalTrials.gov, identifier NCT04696653.

## 1 Introduction

Cardiovascular disease (CVD) is the leading cause of premature mortality in populations with serious mental illness (SMI), such as schizophrenia or bipolar disorder (1, 2). Populations with SMI have a high prevalence of CVD risk factors, including hypertension, diabetes, dyslipidemia, obesity, and tobacco use (3–6). However, this population with SMI is also less likely to receive evidence-based care for these CVD risk factors (3–6). Use of psychotropic medications further elevates their CVD risk (7, 8).

Within the United States, CVD risk factor management has mainly occurred in primary care. The American College of Cardiology and American Heart Association recommend a team-based care approach to manage CVD risk (9). They outline specific blood pressure, glycemic, and cholesterol goals and recommendations to prevent CVD, particularly for those with multiple risk factors (9, 10). Some recommendations, such as dietary modifications and increasing physical activity pertain to all individuals at risk for CVD. In addition, some medications are recommended across two or more CVD risk factors, such as statin therapy for diabetes and dyslipidemia treatment or ACE inhibitors/angiotensin receptor blockers for diabetes and hypertension treatment (10, 11).

Yet people with SMI are seen frequently in the specialty mental health settings (12). This historical separation, or siloing of care, has led to patients falling through the cracks in the U.S. health care system (12). Initial efforts to integrate specialty mental health and physical health settings were based in primary care (e.g. Primary Care Medical Home) (13, 14). In 2010, the U.S. Congress passed the Affordable Care Act, a comprehensive bill aimed at reforming healthcare delivery. One provision allowed the public health insurance, Medicaid, to fund a health home waiver program. This “health home” program was implemented across 18 states and the District of Columbia and allows specialty mental health centers to bill for care coordination and care management for physical health conditions, such as CVD and CVD risk reduction (15). Clinical trials using nurse care managers to conduct care coordination and population health strategies demonstrated positive outcomes in cardiometabolic care, measured as a composite score, and in cholesterol management (16–18). However, real-world behavioral health homes demonstrated limited success in improving outcomes, such as glycemic control (13, 16, 19, 20). Prior literature has reported that mental health staff express uncertainty of what evidence-based CVD risk factor care is, as many come from a clinical background in mental health and not CVD risk factor care (13, 21). The Medicaid health home waiver program provided financing option but did not provide knowledge and system-based resources for to put CVD risk factor care and care coordination into practice. For example, community mental sites have reported lack structured protocols to directly address CVD risk factor care (13, 21). In addition, a majority of mental health staff perceive that improving physical health should be part of their organization’s mission. However, the day to day work of delivering CVD risk factor falls to individual nurse care managers who often operate as a solo provider and not wider teams within the organizations (13, 21).

Team-based strategies have been successful in improving guideline-concordant care delivery in complex medical settings, such as reducing hospital-acquired infections within intensive care units, increasing uptake of preventive cancer screenings, and increasing vaccine uptake in outpatient clinics (22–25). These teams were comprised of individuals who were involved in all aspects of patient care, from the front line administrative assistants, to medical assistants, to social workers, and to nurses and physicians. Yet less is known about levering multi-disciplinary teams within in specialty mental health settings to improve physical health. In specialty mental health settings, patient care includes care managers, social workers, rehabilitation therapists, nurses, and psychiatrists (26, 26). This study sought to address CVD risk reduction in specialty mental health settings using a team-based quality improvement approach. Specifically, we pilot tested an adapted team-based implementation strategy, the Comprehensive Unit-Based Safety Program (CUSP) to support delivery of evidence-based care for diabetes, hypertension, and dyslipidemia. These three CVD risk factors were chosen as they have overlapping recommendations for lifestyle changes and medication management.

## 2 Methods

### 2.1 Study design

We conducted a single arm pilot study of a quality improvement (a QI) implementation strategy using a pre/post observational design between February 2021and April 2023. The Institutional Review Board at Johns Hopkins University approved the protocol. All site staff participants provided written/oral consent after receiving a complete description of the study. Client data was collected under a waiver of documentation of consent. The trial was registered at clinicaltrials.gov (#NCT04696653). No deviations from the protocol occurred.

### 2.2 Study setting

We enrolled four psychiatric rehabilitation programs who have implemented Medicaid-supported behavioral health home programs in Maryland. Psychiatric rehabilitation programs serve clients with significant functional impairment because of their mental illness and provide outpatient mental health and psychosocial services, including vocational training, employment, and mental health case management. These programs focus on helping clients work towards self-identified goals and to live more independently within the community (27). Staff at psychiatric rehabilitation programs included case managers, rehabilitation therapists, social workers, front-line administrative assistants, and nurses. Often, the case managers will liaise with psychiatrists. Within Maryland, psychiatrists refer patients to the psychiatric rehabilitation programs but are not directly involved in the day-to-day activities at the programs. Health home services for care coordination and care management for physical health conditions are billed on a per member, per month basis and are in addition to psychiatric rehabilitation services. Health homes may employ a nurse care manager (21).

### 2.3 Implementation strategy and evidence bundle

#### 2.3.1 Comprehensive unit-based safety program implementation strategy

This study used the Comprehensive Unit Based Safety Program (CUSP) implementation strategy (28) to directly improve how programs systematically deliver evidence-based CVD risk factor care, a previously identified gap within behavioral health home programs (13). CUSP teams used an evidence bundle to identify what actions they were (or were not) doing for delivery of guideline-concordant care. Description of the evidence-bundle is described below.

The goal of CUSP is to provide healthcare teams with a structured process and toolkit to identify and to address barriers that interfere with their ability to delivery evidence-based care and to improve how they work together. It also seeks to foster a culture of continuous quality improvement (QI) and provider self-efficacy within inpatient and outpatient QI work (23, 28, 29). In this study, training was delivered at the site level and included provider training, external expert facilitation, and CUSP’s quality improvement (QI) process (30). CUSP’s core steps are: 1) all staff receive training in the science of QI; 2) CUSP teams create processes to identify barriers to evidence-based care; 3) CUSP teams create and implement processes for engaging senior leadership; 4) CUSP teams address barriers; and 5) CUSP teams work to improve teamwork and communication (28, 30). Steps 2-5 are conducted in parallel.

In this study, core CUSP components were implemented in alignment with the model. Teams identified barriers that presented challenge to their organization in delivering CVD risk factor care, key leaders within their organization, and potential strategies to move the project forward (30). CUSP materials were adapted to the mental health setting to align with existing organizational culture, workflow, and roles and responsibilities. For example, a CUSP tool for the inpatient setting (“learning from defects”) asks teams to identify causes and potential solutions for “defects” that can lead to adverse patient safety events (30). Language was adapted to “learning from challenges” to reflect anticipated barriers in the outpatient setting.

#### 2.3.2 Evidence bundle

The intervention focused on hypertension, diabetes, and dyslipidemia in populations with SMI as these CVD factors share overlapping care processes. Compared with other CVD risk factors, hypertension, diabetes, and dyslipidemia have overlapping lifestyle counseling and medication management recommendations. Medication management is a key area where care coordination is needed across specialty mental health and primary care (12, 13). Care coordination was emphasized as it fit a team-based intervention and is a Medicaid-reimbursed service in behavioral health homes. Some cardiovascular risk factors, such as tobacco use, were not included as counseling approach (e.g. identifying triggers for smoking, coping strategies to avoid cigarette use) are unique to tobacco use. Moreover, medications for smoking cessation (e.g. varenicline) are distinct from medications used for hypertension, diabetes, and dyslipidemia.

An evidence bundle was assembled to outline what constitutes guideline-concordant care for hypertension, diabetes, and dyslipidemia. Prior CUSP interventions have used an evidence bundle to guide CUSP teams who may not have known the guidelines (23, 28, 29). Assembly of the bundle was conducted using the TRiP framework, which outlines how to synthesize evidence and then implement clinical practice (**Appendix**) (31).

The bundle summarized evidence-based screening practices and management target goals according to the American College of Cardiology/American Heart Association (ACC/AHA), and American Diabetes Association (ADA) (9, 11, 32). It also included best practice guidelines for populations with SMI, according to the American Psychiatric Association and literature, such as screening for metabolic sequalae of antipsychotic medications and caring for individuals with cognitive dysfunction (33–35). The bundle included best practices for population health, care management, and care coordination as cross-cutting care processes according to the Agency for Healthcare Research and Quality (AHRQ) and Patient Centered Medical Home criteria (36, 37). Population health identifies and track changes to a group’s health over time (38). Care management addresses an individual patient’s health needs, while care coordination aligns health services with the individual’s needs (37). Bundle assembly was led by two general internists, a nurse with experience in caring for patients with SMI, and an expert in motivational interviewing, all part of the study team. It was reviewed for clinical accuracy and feedback by three clinical experts (external members of study team) and for acceptability and feedback by two health home nurse care managers (external members of study team).

#### 2.3.3 Study phases

We implemented CUSP in three phases: pre-implementation (2-months), implementation (12-months), and sustainment (3-months), for a total of 17 months at each site (**Table A1**) (30). Each site formed an interdisciplinary CUSP team with staff from the psychiatric rehabilitation programs and behavioral health homes.

In the 2-month pre-implementation phase, CUSP teams received training in advanced CUSP processes (four 1-hour sessions), evidence-based care management and coordination for CVD risk factors (two 2-hour sessions), motivational interviewing (two 2-hour sessions), and sustaining CUSP teams (one 1-hour session). During training, CUSP team members received the evidence bundle by both digital and print format. The training reflected the content of the evidence bundle. The motivational interview training focused on how to engage clients with their own care needs for CVD risk factors (e.g. self-management strategies for diabetes). It was tailored to staff working in specialty mental health clinics (39). Staff (CUSP and non-CUSP team members) filled out pre-implementation surveys. CUSP members then identified initial implementation barriers and reviewed pre-implementation survey data.

During the 12-month implementation phase, CUSP teams implemented the CUSP process. They met monthly as a team and with a CUSP expert facilitator from the study team. CUSP teams held a training on QI (1 hour duration) for staff. Sites selected which CVD risk factors to address, which barriers and how many potential solutions or practice changes to address at any given time based on their site’s available resources. They also identified which clients had diagnosis of hypertension, diabetes, or dyslipidemia (e.g. size of population with a CVD risk factor), what clinical data they had or needed to obtain on these clients’ CVD risk factor, and then who they needed to coordinate care with for a client (e.g. primary care physician). They then implemented activities to address self-identified barriers to evidence-based CVD risk factor coordination.

Finally, in the 3-month sustainment phase, the CUSP teams continued to address self-identified barriers but did not meet with the facilitator.

### 2.4 Measures

Our primary outcomes focused on changes in a) QI culture at the organizational level and b) provider self-efficacy to delivery CVD care coordination during the 12-month implementation phase. Our secondary outcomes focused on changes in c) acceptability, appropriateness, and feasibility of CUSP implementation strategy and use of the evidence bundle for CVD risk factor and d) the percentages receipt of guideline-concordant care for hypertension, diabetes, and dyslipidemia for clients enrolled in behavioral health homes. Data was collected during the pre-implementation phase and end of the implementation phase.

#### 2.4.1 Quality improvement culture

We used a modified Survey on Patient Safety, a validated thirty-four item instrument that measures QI culture across nine domains (40). It examines teamwork, supervisor promotion of QI work, organizational learning, management support for QI, feedback and communication, communication openness, reporting mistakes, staffing capacity, and overall capacity for QI. On a 5-point Likert scale, higher scores indicate organizations that are more conducive to QI activities. All staff (CUSP team members and non-CUSP team members) were asked to complete this survey. As CUSP team members received a longer training session on QI, responses were stratified by CUSP team membership status.

#### 2.4.2 Provider self-efficacy

We used a modified Compeau and Higgins’ scale, on a nine-item instrument, to measure provider self-efficacy to coordinate evidence-based care for hypertension, hyperlipidemia, or diabetes (41). On a 10-point Likert scale, higher scores indicate greater self-efficacy. As only CUSP team members were given training on hypertension, hyperlipidemia, and diabetes, only CUSP team members were asked to complete this survey.

#### 2.4.3 Acceptability, appropriateness, feasibility

We used the following validated survey measures: Acceptability of Intervention Measure (AIM), Intervention Appropriateness Measure (IAM), and Feasibility of Intervention Measure (FIM) (42). Each measure includes four items using a 5-point Likert scale, with higher scores signifying greater acceptability, appropriateness, or feasibility. Only CUSP team members were asked to complete these surveys on the CUSP strategy and use of the evidence bundle.

#### 2.4.4 Evidence-based care for CVD risk factors

Finally, we measured the percentage of individuals who reached the recommended evidence-based targets for each CVD risk factor as recommended by the U,S. guidelines from the ACC/AHA and ADA (9, 11, 32). The ACC/AHA recommend a target blood pressure of less than 130/80 mm Hg (strict goal) or less than 140/90 mm Hg (more lenient goal) among individuals with hypertension. The ADA recommends a target hemoglobin A1c of less than 7% (strict goal) or hemoglobin A1c of less than 8% (lenient goal) among individuals with diabetes. Finally, the ACC/AHA recommends that individuals be prescribed a statin drug as a first-line medication for treatment of hyperlipidemia and a target low density lipoprotein (LDL) <100 mg/dL (strict goal) or <130 mg/dL (lenient goal).

### 2.5 Data collection

Survey data was collected using REDCap. Staff and CUSP team members were surveyed during the pre-implementation phase and at the end of the 12-month implementation phase. Sites self-reported client demographics and presence of CVD risk factor from records, and if had capability, measured blood pressure. One site had access to existing health system data; all other sites requested notes and laboratory data from external primary care clinics. Consistent with population health practice (38), sites tracked clients’ blood pressure, hemoglobin A1c, and lipid panel results across the implementation phase. Client data was provided to the study team during the transition from the pre-implementation phase to implementation phase as pre-implementation data and data available at the end of the 12-month implementation phase was provided as post-implementation data.

### 2.6 Statistical analysis

We used mixed-effect repeated-measure regression models to measure changes in outcomes that used Likert scales (QI culture and provider self-efficacy, and perceptions of acceptability, appropriateness, feasibility) and generalized estimating equations (GEE) to measure changes in probability of clients receiving guideline-concordant care. For both approaches, the pre-post comparisons were evaluated with the corresponding 95% confidence intervals (CIs) derived while adjusted for study sites. The QI culture outcome model also included an additional binary covariate indicating participation on the CUSP Team (Yes/No). For the mixed-effects modeling, an unstructured variance-covariance model was used for the longitudinally measured outcomes to allow different outcome variance at different timepoints and address the within-subject (staff) correlation between the repeated outcomes across timepoints. For GEE analyses, an exchangeable working correlation was used, and the sandwich-based robust estimates were used for statistical inferences. Available data for all enrolled participants was included in these modeling-based analyses, including those with only pre or post data (e.g., due to staff turnover).

To explore for site specific effects, mixed-effects models included additional cross-product interaction terms between study site variables and the timepoint variable, all as fixed effects, were used (43). For the exploratory analyses using the GEE approach employing visit by site interactions, the sites with identical outcome responses (e.g., 100% yes) pre or post intervention within site were excluded due to numerical challenge of model convergence. These sites were noted and described in the results with descriptive statistics. As a sensitivity analysis, overall effect estimates were also modeled using only data from sites that could be included in the model for site-specific effect estimation. We also reported effect size based on Cohen’s d statistic, with interpretation of d in 0.00-0.19 as very small; 0.20-0.49 as small; 0.50-0.79 as medium; and d ≥ 0.80 as large effect (43).

## 3 Results

### 3.1 Study sites, staff, and client participants

Participating sites provided virtual care or hybrid of virtual and in-person care in the first six months of the study. As COVID-19 pandemic restrictions evolved, one site transitioned to to fully in-person care, one site remained fully virtual, and two sites delivered care in a hybrid format (**Table 1**).

**Table 1:** Characteristics of participating sites, staff, and client populations. All data is pre-implementation unless otherwise noted.

CHARACTERISTIC	ALL SITES	SITE 1	SITE 2	SITE 3	SITE 4
Site Characteristics					
Services Provided	—	Outpatient	Outpatient, Residential	Outpatient, Residential	Outpatient
Geographic Setting	—	Urban	Suburban	Suburban	Urban, Suburban
Service Modality^1^	—	Hybrid	Hybrid	Hybrid	Telehealth
Access to shared electronic health record or coordination tool with primary care	—	Yes	No	No	No
Staff Characteristics					
	(N=85)	(N=26)	(N=16)	(N=21)	(N=22)
Age, years – mean (SD)	35.2 (11.9)	35.0 (11.9)	40.7 (15.6)	36.2 (8.9)	30.4 (10.1)
Female, n (%)	74 (87.1)	20 (76.9)	15 (93.8)	19 (90.5)	20 (90.9)
Education, n (%)					
< College	8 (9.4)	3 (11.5)	1 (6.3)	4 (19.0)	0 (0.0)
Bachelor’s degree	56 (65.9)	18 (69.2)	8 (50.0)	12 (57.1)	18 (81.8)
Master’s degree or higher	21 (24.7)	5 (19.2)	7 (43.8)	5 (23.8)	4 (18.2)
Employed full time, n (%)	77 (90.6)	26 (100.0)	13 (81.3)	21 (100.0)	17 (77.3)
Turnover during study, n (%)	19 (22.4)	6 (23.1)	4 (25.0)	5 (23.8)	4 (18.2)
Staff Roles, n (%)					
Director/Manager^2^	20 (23.5)	5 (19.2)	3 (18.8)	9 (42.9)	3 (13.6)
Nurse	5 (5.9)	1 (3.8)	3 (18.8)	0 (0.0)	1 (4.3)
Direct Care- Mental Health^3^	48 (56.5)	17 (65.4)	5 (31.3)	9 (42.9)	17 (77.3)
Direct Care- Other^4^	12 (14.2)	3 (11.5)	5 (31.3)	3 (14.3)	1 (4.5)
CUSP team roles, n (%)	(n=19)	(n=6)	(n=6)	(n=3)	(n=4)
Director/Manager	6 (31.6)	2 (33.3)	2 (33.3)	1 (33.3)	1 (0.25)
Nurse	4 (21.1)	1 (16.7)	2 (33.3)	0 (0.0)	1 (0.25)
Direct Care	9 (47.4)	3 (50.0)	2 (33.3)	2 (66.6)	2 (0.5)
Client Characteristics^5^					
	(N=442)	(n=104)	(n=83)	(n=149)	(n=106)
Psychiatric Diagnosis, n (%)					
Bipolar disorder	112 (25.3)	18 (17.3)	12 (14.5)	38 (25.5)	44 (41.5)
Major depression	130 (29.4)	39 (37.5)	12 (14.5)	37 (24.8)	42 (39.6)
Schizophrenia	200 (45.2)	47 (45.2)	59 (71.1)	74 (49.7)	20 (18.9)
CVD Risk Factor, n (%)					
Hypertension	189/434 (43.5)	46 (44.2)	43 (51.8)	50/148 (33.8)	50/99 (50.5)
Diabetes	123/431 (28.5)	24 (23.1)	29 (34.9)	34/148 (23.0)	36/96 (38.0)
Dyslipidemia	166/406 (26.1)	38 (36.5)	34/82 (41.5)	53/146 (36.3)	41/74 (55.4)
BMI>=25.0 kg/m^2^	337/402 (83.8)	84 (80.8)	70/79 (88.6)	100/122 (82.0)	83/97 (85.6)
Current smoker	186/432 (43.1)	48/103 (46.6)	37 (44.6)	60/147 (40.8)	41/99 (41.4)
>=3 risk factors	188/441 (42.6)	40/104 (38.5)	41/83 (49.4)	51/148 (34.5)	56/103 (54.4)

^1^Hybrid service delivery: telehealth and in-person care.

^2^Site director, HR director, health home director, county manager, CEO, clinical supervisor, support staff supervisor.

^3^Mental health care coordinator and treatment plan specialist, community rehabilitation specialist, case manager, rehabilitation direct care coach, intake coordinator, PRP coordinator.

^4^Residential staff, employment specialist, clerical, driver.

^5^Clients with any CVD risk factor or psychiatric diagnosis data available at pre-implementation; denominators given if client-level data unavailable for specific CVD risk factor.

Across all sites, 85 staff members participated, of which 19 were a part of a CUSP team and 66 were not a part of a CUSP team. Sites had 16-26 staff members, and CUSP teams comprised of 3-6 members. Staff were predominately female-identifying (74%), white race (72%), college-educated (91%), and employed full-time (91%) (**Table 1**, **Appendix**). Staff worked as mental health coordinators and rehabilitation therapists (57%) or as front-line providers, such as employment specialists or residential program staff (14%). Three out of four sites had nurses who were affiliated with the behavioral health home and participated on CUSP teams. Site directors or managers participated in CUSP teams at all sites.

The pilot included 442 clients, and individual sites served 83-149 clients (**Table 1**, **Appendix**). Sites reported their client population had a mean age 47.5 years (SD=14.2 years), were 53% male, 51% white race and 45% Black race. Approximately 45% of clients were diagnosed with schizophrenia, 25% had bipolar disorder, and 29% had major depressive disorder, with the prevalence of each mental health diagnoses varying across sites. Approximately 33% of clients had a history of alcohol or substance use disorder, and 68% of clients received disability. At baseline. At pre-implementation, there were 44% of clients with a history of hypertension, of whom 70% had uncontrolled hypertension. Of the 29% of clients with a history of diabetes, 42% had poor glycemic control. Of the 26% of clients with a history of dyslipidemia, 21% had elevated cholesterol levels. In addition, 76% clients were overweight or obese (body mass index>25) and 43% of clients smoked tobacco.

### 3.2 Quality improvement culture

CUSP and non-CUSP staff reported on how they perceived organizational QI culture between pre-implementation (N=84) and post-implementation (N=64). Staff perceived no difference across time (3.8 [SD=0.7] vs. 3.8 [SD=0.6]; estimate change 0.0 [95% CI: -0.2-0.1]. The effect size was very small, measured by Cohen’s d, was d=-0.05 (**Table 2**). Responses were similar between CUSP and non-CUSP team members and across sites. In addition, no differences across the 12-month measurement period were observed when we looked at sub-measures of quality improvement culture (**Table A4**). Lowest ratings were given to the categories of staffing capacity (2.9 [SD=0.9] vs. 2.8 [SD=2.9; estimate change -0.2 [95% CI: -0.4- 0.0]; d=-0.19) and openness of communication (3.5 [SD=0.8] vs. 3.5 [SD=0.8]; estimate change 0.1 [95% CI: -0.1-0.3]; d=0.11).

**Table 2:** Summary of changes across the 12-month implementation period for primary outcomes: quality improvement culture and perceived provider self-efficacy for cardiovascular disease risk factor coordination.

	Pre		Post		Change		
	n	M (SD)	n	M (SD)	Estimate (95% CI) ^3^	Cohen’s d	Effect size ^4^
Quality Improvement Culture ^1^							
All participants	84	3.8 (0.7)	64	3.8 (0.6)	0.0 (-0.2, 0.1)	-0.06	Very small
Non-CUSP only	62	3.9 (0.6)	45	3.8 (0.6)	0.0 (-0.2, 0.1)	-0.06	Very small
CUSP Team	22	3.5 (0.9)	19	3.6 (0.7)	0.0 (-0.2, 0.1)	-0.06	Very small
Self-Efficacy to Coordinate Care ^2^							
Hypertension	22	6.0 (2.3)	19	7.1 (1.2)	1.0 (0.1, 1.9) *	0.44	Small
Dyslipidemia	22	5.9 (2.4)	19	7.0 (1.4)	1.0 (-0.1, 2.1)	0.43	Small
Diabetes	22	6.2 (2.6)	19	7.3 (1.4)	1.0 (0.0, 1.9) *	0.37	Small

*p<0.05.

^1^Quality Improvement Culture scale: 1 (needs improvement) to 5 (strong culture). Completed by all staff and CUSP team members.

^2^Health Home Self-Efficacy scale: 0 (no self-efficacy) to 10 (high self-efficacy). Completed by CUSP team members.

^3^Estimates derived from outcome specific mixed-effects repeated measures regression models utilizing all available data, with fixed effects for study site and CUSP team status for the Quality Improvement Culture. For the Self-Efficacy the model is the same but without effects for CUSP team status as all respondents are a part of the CUSP team.

^4^Effect size interpretation^43^.

### 3.3 Provider self-efficacy

CUSP team members reported on perceived self-efficacy for coordinating for each CVD risk factor from pre-implementation (N=22) to post-implementation (N=19). Perceived self-efficacy increased significantly for two topics: hypertension (6.0 [SD=2.3] vs. 7.1 [SD=1.2]; estimate change 1.0; (95% CI: 0.2-1.8); d=0.44, small effect) and diabetes (6.2 [SD=2.6] vs. 7.3 [SD=1.4]; estimate change 1.0 [95% CI: 0.1-2.0]; d=0.4, small effect). Self-efficacy also increased for dyslipidemia management but did not reach statistical significance (5.9 [SD=2.4] vs. 7.0 [SD=1.4]; estimate 1.1 [95% CI: 0.0-2.1]; d=0.44, small effect). Self-efficacy scores to coordinate care were driven by large effect sizes at Sites 1 and 3, as measured by Cohen’s d (**Table A3**).

### 3.4 Acceptability, Appropriateness, and Feasibility

CUSP team members also reported pre-implementation (N=19) and post-implementation (N=19) on the acceptability, appropriateness, and feasibility of the CUSP implementation strategy and evidence-bundle outlining CVD risk factor care guidelines (**Table 3**). Scores for the acceptability of the CUSP implementation strategy did not change significantly over time (4.4 [SD=0.5] vs. 4.2 [SD=0.6]; estimate change -0.2 [95% CI: -0.6-0.1], d= -0.49). Similar scores were observed for appropriateness and feasibility of the CUSP strategy. Scores for acceptability of the evidence bundle also did not change significantly across time (4.4 [SD=0.5] vs. 4.2 [SD=0.5]; estimate change -0.3 [95% CI: -0.6-0.0], d= -0.59). Scores were similar for appropriateness and feasibility of the evidence bundle (**Table 3**).

**Table 3:** Summary of outcomes of acceptability, appropriateness, and feasibility of the CUSP implementation strategy and evidence bundle to improve delivery of cardiovascular risk factor care coordination.

	Pre		Post		Change		
	n	M (SD)	n	M (SD)	Estimate (95% CI) ^2^	Cohen’s d	Effect size ^3^
Acceptability, Appropriateness, and Feasibility ^1^							
CUSP Strategy							
Acceptability	19	4.4 (0.5)	19	4.2 (0.6)	-0.2 (-0.5, 0.1)	-0.41	Small
Appropriateness	19	4.4 (0.5)	19	4.2 (0.7)	-0.2 (-0.6, 0.1)	-0.51	Medium
Feasibility	19	4.3 (0.4)	19	4.2 (0.6)	-0.1 (-0.4, 0.2)	-0.28	Small
Evidence Bundle							
Acceptability	19	4.4 (0.5)	19	4.2 (0.5)	-0.2 (-0.6, 0.1)	-0.48	Small
Appropriateness	19	4.3 (0.5)	19	4.2 (0.7)	-0.1 (-0.4, 0.2)	-0.30	Small
Feasibility	19	4.3 (0.4)	19	4.2 (0.6)	-0.1 (-0.4, 0.2)	-0.28	Small

^1^Acceptability, Appropriateness, and Feasibility scale: 1 (not acceptable/appropriate/feasible) to 5 (highly acceptable/appropriate/feasible). Completed by CUSP Team.

^2^Estimates derived from outcome specific mixed-effects repeated measures regression models utilizing all available data, with fixed effects for study site.

^3^Effect size interpretation^43^.

### 3.5 Receipt of evidence-based care for CVD risk factors

Hypertension: Across all sites, the likelihood of having blood pressure strictly controlled at <130/80 mm Hg did not change over time (OR 1.4, 95% CI: 0.9-2.2) (**Table 4**). Clients at site 1 were more likely to have achieved a more lenient goal of blood pressure < 140/90 mm Hg after the intervention (OR 3.0, 95% CI: 1.2-7.8); however, this was not observed at other sites (**Table A7**). In addition, no difference was seen over time in the percentage of the client population with an available blood pressure in the prior 12-months.

**Table 4:** Number and percentage of clients with hypertension, diabetes, and dyslipidemia with available clinical data and those who have reached the recommended target for blood pressure control, glycemic control as measured by hemoglobin A1c, and cholesterol control across the 12-month implementation stage (pre-implementation vs. post).

	Pre	Post	Change
	n (%)	n (%)	Odds Ratio (95% CI) ^1^
Hypertension			
Identified ^5^	189	203	
Available BP data	166 (88)	186 (92)	1.8 (1.0, 3.3)
BP < 130/80 ^2^	41 (25)	59 (32)	1.4 (0.9, 2.2)
BP within 1 year	156 (94)	168 (90)	0.7 (0.3, 1.5)
Diabetes			
Identified ^5^	123	120	
Available HgbA1c data	92 (75)	99 (83)	1.8 (1.2, 2.6)
HgbA1c < 7% ^3^	53 (58)	55 (56)	1.0 (0.7,1.4)
HgbA1c within 1 year	81 (88)	83 (84)	0.3 (0.1, 0.6)
Dyslipidemia			
Identified ^5^	166	184	
Available lipids data	131 (79)	133 (72)	1.2 (1.0, 1.6)
LDL-C < 100 mg/dl ^4^	70 (53)	75 (56)	1.4 (1.1, 1.9)
Lipids within 1 year	108 (82)	78 (59)	0.3 (0.2, 0.6)

BP, Blood pressure; HgbA1c, Hemoglobin A1c; LDL-C, Low density lipoprotein cholesterol

^1^Odds ratio was derived from binomial generalized estimating equations (GEE) models utilizing all available data from all four study sites and adjusting for study site.

^2^Strict control for blood pressure is defined as <130/80 mmHg per national guidelines from the American College of Cardiology/American Heart Association.

^3^Strict glycemic control is defined as HgbA1c <7% by the American Diabetes Association.

^4^Strict cholesterol control is defined as LDL-C <100 mg/dl by the American College of Cardiology/American Heart Association.

^5^Clients identified by health home nurse as having the given cardiovascular disease (CVD) risk factor. Differences in subpopulation size with a given CVD risk factor reflect clients enrolling and disenrolling at participating sites and sites/primary care identifying new medical conditions. Only clients with the CVD risk factor were included for each CVD risk factor.

Diabetes: Across all sites, we observed no change in the likelihood of clients having achieved strict glycemic control (Hemoglobin A1c < 7%) (OR 1.2; 95% CI: 0.7-2.2) or more lenient glycemic control (Hemoglobin A1c < 8%) (OR 1.2; 95% CI: 0.7-2.2) (**Table A7**). There was also no difference in the percentage of clients who had a hemoglobin A1c value available in the prior 12-months.

Dyslipidemia: Across all sites, clients were more likely to have an LDL-C value of < 100 mg/dl after the intervention (OR 1.4; 95% CI: 1.1-1.9) (**Table 4**). However, only clients at Site 1 had an increased likelihood in reaching this cholesterol measure (OR 1.8; 95% CI-1.1-2.9) (**Table A7**). Notable, Sites 2 and 4 had available cholesterol laboratory data for 54-68% of their client population.

### 3.6 Activities of CUSP team

CUSP teams held monthly meetings at least 80% of the time during implementation phase (**Table 5**), but only two sites held formal meetings during the sustainment phase (when external facilitator was not present). All sites addressed at least one CVD risk factor. The three sites that delivered in-person care addressed hypertension.

**Table 5:** Activities of CUSP team during the 12-month implementation and 3-month sustainment phases.

ACTIVITIES	SITE 1	SITE 2	SITE 3	SITE 4
CUSP monthly meetings held, n (%)				
Implementation phase	12 (100.0)	12 (100.0)	11 (91.7)	10 (83.3)
Sustainment phase	3 (100.0)	3 (100.0)	0 (0.0)	0 (0.0)
CVD Risk Factors Addressed				
Hypertension	X	X	X	
Diabetes	X	X		X
Dyslipidemia		X	X	
Tobacco use^1^		X		
Processes Developed and Implemented to Address Barriers to CVD Risk Factor Coordination				
Solicit Feedback within Organization				
Solicited feedback to decrease client CVD risk	X^2^	X^2^	X	X
Opened CUSP team to all direct care staff	X			
Education				
Delivered CVD risk factor education to clients		X		
Delivered CVD risk factor education to staff		X	X	X
Implemented external certification for staff.^3^		X		
Staff Capacity to Coordinate Care				
Shared medical appointment calendar	X	X		X
Obtained result viewing approval from external laboratory network				X
Standardized process to obtain records	X			X

^1^Not target CVD risk factor of implementation intervention.

^2^Implemented during the sustainment phase.

^3^Obtained external certifications on smoking cessation.

All CUSP teams sought staff-wide feedback about how to improve CVD risk factor coordination during the implementation phase, and two teams conducted an additional survey during the sustainment phase. Site 1 opened meetings to all staff and received feedback on CUSP activities from direct care staff. Three teams also delivered CVD risk factor education for staff and/or clients. Site 3 implemented this staff-wide training twice yearly, while Site 2 sponsored staff to become certified in tobacco cessation counseling.

Finally, CUSP teams created processes to improve intra-organization communication and to build staff capacity for coordination for CVD risk factor care. For example, direct care staff did not have a standardized process to communicate when clients had medical appointments to the nurse. Three teams implemented a shared electronic calendar of client appointments between staff and the nurse(s). This process took three to eight months and required buy-in from administrators for technology resources, training, and workflow changes.

Sites also identified differential access to clients’ health data, and this lack of information was a barrier that they sought to address. They felt that they did not know which clients were receiving guideline-concordant care and which clients to prioritize for CVD risk factor coordination. Site 1 had access to electronic health data for CVD risk factors as many of their clients saw primary care physicians within a shared electronic health record. However, they did not have a system to obtain the data. Site 4 had no access to existing laboratory data or a system to know when clients had last seen a primary care physician. Thus, these two sites implemented a new standardized workflow for direct care staff to obtain laboratory data and health records from primary care physicians and specialists. This was incorporated as part of client intake and meetings with case managers. Site 4 also implemented a process where clients could grant permission for their mental health team to access results directly from an external clinical laboratory network. This process allowed the nurse to monitor chronic disease measures (e.g. hemoglobin A1c). Both sites then planned to use the information to coordinate appointments for clients who had not seen a primary care physician in over a year or who had a CVD risk factor that was poorly controlled.

## 4 Discussion

In this pilot, four community mental health behavioral health home programs that served populations with SMI implemented a team-based quality improvement strategy focused on CVD risk factor care quality. We observed a medium effect increase in provider self-efficacy score to coordinate CVD risk factor care but no significant change in the organizations’ QI culture. In secondary outcomes, use of an evidence bundle and the team-based QI strategy had high scores for acceptability, appropriateness, and feasibility at the start and end of the implementation, phase, but no interval change was observed.

This pilot study’s findings reflect the challenges of implementation within specialty mental health settings, addressing provider and organizational behavior and culture within a 12-month time frame and the complexity of the intervention. All sites served a high-need, vulnerable population with SMI. The client population had high prevalence of disability (likely because of impairment from mental illness) who had been referred by their psychiatrist for vocational training and community-support services. While the study team did not collect data on socioeconomic status, clients likely had low income as they were enrolled in public Medicaid insurance (health home programs were supported by Medicaid). Moreover, our findings echo prior literature that Medicaid-funded health home programs have struggled with implementing CVD risk factor management due to lack of knowledge and resources (23, 28, 29). None of the sites had existing population-based approach for managing clients’ CVD risk factors where they knew which groups of clients had which CVD risk factors. No sites also had pre-existing resources to identify whether clients had achieved CVD risk factor control. Rather, they relied on addressing CVD risk factors on an *ad hoc* basis and if an individual client came to the nurse with a question. We also observed the limited resources and staff turnover at these organizations, which further suggested why organizations struggled to deliver services.

In the intervention, CUSP teams were charged with developing and initiating new processes for coordination of CVD risk factor care for their client population with SMI. By doing so, they started shifting CVD risk factor care from the sole responsibility of a nurse to a team-based care model. Three out of four sites focused on improving how teams worked together and highlighting infrastructure barriers. The prioritized addressing foundational team processes (intra-team communication, obtaining client population data, education) that are needed to support CVD risk factor care. These site-identified barriers mirror the barriers that have been reported in other behavioral health home programs (13). This suggests that community mental health sites need time and resources to put key infrastructure and team processes in place before they can successfully focus on improving specific CVD risk factor care processes.

Our study is the first to pilot CUSP in specialty mental health programs and to use CUSP for a topic (for CVD risk factors) not routinely addressed by providers in these settings. CUSP implementation strategy was chosen as it fit into a team-based care model, which is the underlying model at psychiatric rehabilitation programs. This may help to explain why sites gave high ratings to the acceptability, appropriateness, and feasibility of the CUSP implementation strategy. Prior CUSP implementation projects have occurred in acute care or primary care and were focused on clinical measures that were within the team’s existing scope of practice (e.g., prevention of line-associated infections in acute care) (23, 28, 29). Prior to this study, no behavioral health home site had previously focused on QI, and improvement in QI culture – which exists at the unit, department, and system levels and is often referred to as “the way we do things here” (44) - is expected over two to three years (45–47). It takes time for culture change to occur and requires administrative support (23). Our results are consistent with this literature. While there is a natural inclination to look for change in any intervention study, this data provides meaningful baseline information about the quality improvement culture at community mental health sites for future studies. In addition, no behavioral health home site had previously focused on QI. In addition, the composite score and responses to individual QI survey questions provide insight about specific areas of strengths and weaknesses (48).

Importantly, our findings reflect similar organizational drivers that have been described in implementation of behavioral health homes and the wider implementation science literature. The sites that saw the greatest increase in self-efficacy to coordinate CVD risk factors had regular support/buy-in from leadership (Sites 1 and 3), greater access to technology (Site 1), and less staff turnover (Sites 1 and 3).

One possible organizational driver is the need for buy-in from organizational members, from top to bottom. CUSP focuses on engagement of staff and senior leadership because leadership engagement is critical for successful implementation and sustainment (30, 49). Each site was asked to have at least one director or manager on the CUSP team. However, only Site 1 had a director regularly attend CUSP meetings. Site 3 had regular check-ins with site leadership and a manager served as the CUSP team lead. Other sites had managers with differing levels of decision-making power. In practice, this translated to teams waiting for administrative approval and delays in implementing activities, such as the shared calendar. Future efforts to build upon this work will need continued support from leadership to address workflow processes and organizational culture. Moreover, CUSP is designed to be a longitudinal strategy for healthcare settings. Yet only half the sites held CUSP meetings during the 3-month sustainment period. The study team provided facilitation support for CUSP strategy during the 12-month intervention period but not during the sustainment period. Future work may be needed on whether additional facilitation support and what type of support is needed to sustain CUSP teams within mental health settings. The study team also did not offer additional incentives for staff to participate in the CUSP team and it was up to CUSP teams to incentivize staff participation for specific activities. Part of the CUSP team’s role is to identify how to engage their colleagues and address practice changes based on their site’s available resources. We did not collect specific data on whether sites offered incentives or not, and future studies may wish to examine how successful mental health sites engaged with staff and leadership. This information will be helpful to understand how to sustain CUSP teams in community mental health settings.

Sites also had different available resources, which may have influenced implementation and sustainability. For example, sites differed on technology, which is a known facilitator (or barrier) to implementation work (13, 49). Site 1 had access to a shared electronic health record with primary care clinicians but at the start of the study, did not know which laboratory measures were relevant to CVD risk factor care. Sites 2, 3, and 4 did not have a shared electronic medical record with primary care clinics and therefore had to request clinical data for their patient populations by fax, email, and phone. This resulted in multiple outreach efforts to primary care offices and greater frustration when no communication was received back. For community mental health sites to be able to conduct population health management and to help coordinate care for populations with SMI, whom they serve, they need accessible data.

In addition, the staff composition, responsibilities, relationships, and morale may have differed across sites, and these internal personnel factors influence success or failure of implementation projects (49). Site 3 was the only site that did not have a nurse onsite and likely a steeper learning curve to implement care processes for CVD risk factors. However, the site team lead was highly engaged, had a manager role and used the evidence-bundle to guide the team. This may also help to explain why the self-efficacy scores rose at Site 3. Sites were asked to have representative staff as part of the CUSP team from across their organization. All four sites had front-line staff and staff who had been with the organization of different lengths of time. Sites with full-time behavioral health home staff (sites 1 and 3) were more successful at obtaining complete client-level data by end of study. This may reflect differential organizational working conditions and structure (50). However, our data did not capture the quality of relationships among staff. Moreover, high staff turnover may have negatively impacted organization culture and ability for teams to implement durable change. Turnover increases individual workloads, slows momentum, and may make process changes harder to sustain (51).

We also gathered data on the implementation outcomes of acceptability, feasibility, and appropriateness and availability and measurement of CVD risk factor clinical data. The relatively high scores at the start and at the end of the study suggest that providers viewed the intervention and QI implementation strategy as a good fit when adopting the study and after implementing it over twelve months (52). While we did not have data on psychiatric medications prescribed to clients across all sites, it is likely that the CUSP teams knew that their clients were prescribed antipsychotic medications, were receiving blood draws, but did not have access to the laboratory values. Therefore, their perception of this intervention is likely consistent with their organizational mission in caring for populations with SMI bu. Consistency with organizational mission is a known facilitator in implementation of physical healthcare delivery within specialty mental health settings (13). However, it is unlikely to have impacted our study’s results.

Our study has several limitations. First, it was conducted in a single state within a small number of psychiatric rehabilitation programs, which may limit its generalizability. Sites also had to agree to participate in a 12-month organizational-wide intervention that focused on addressing physical health in their clients. Sites had 3-6 staff members participate in the CUSP teams, which limited our ability to detect differences over time. No psychiatrists were involved at any site. In Maryland where our study was conducted, psychiatrists refer patients with SMI to psychiatric rehabilitation programs for community services and vocational training. These programs may not be affiliated with their psychiatric clinic. Given the role of psychiatrists in selecting antipsychotic medications that influence CVD risk, future studies may wish to actively include psychiatrists in CUSP team activities.

Second, this study was conducted during the COVID-19 pandemic, which led to virtual meetings for staff and clients. Virtual interactions may also have impacted staff morale and organizational culture. In the second year of the pandemic, sites had differing capacities about resources and time to devote to a complex pilot intervention. Our pre-specified data collection plan in the protocol also did not include collecting detailed data on, so sites’ data did not capture detailed information about sites’ activities. Sites did not have pre-existing services for lifestyle counseling, such as diet and physical activity. We did not collect data on how often obesity management strategies (e.g. diet/exercise counseling, starting Metformin or other adjunctive medication) were offered or received. We also did not collect data on other care coordination process measures, such as referrals or appointments scheduled, attendance rate). This type of data would be helpful in future studies. Future implementation work for this pilot also will need to consider how sites deliver care, as all sites delivered telehealth care with some staff working remotely and its impact on organizational culture and care delivery (53). Third, we had approximately 25% staff turnover across the sites. However, this level of turnover is not dissimilar to what has been reported in the literature prior to the pandemic (54).

Finally, it is likely that this pilot study lacked adequate power to detect large changes in primary outcomes. Primary outcomes were chosen based on prior CUSP studies and reflected the purpose of CUSP to address the quality of care delivered within a given organization (28). Interpretations of QI culture may be also limited by ceiling effect as responses started between 3 or 4 (out of 5). Moreover, by measuring QI culture and its components over time during the pilot study, the study team and participating sites were able to study the approach of implementing CUSP and to identify specific areas of QI culture to target in the future: staff turnover and openness of communication.

Overall, our pilot study observed improvement in self-efficacy to manage CVD risk factor coordination but no significant change in organizational quality improvement culture. The four mental health sites implemented and used the CUSP strategy to focus on improving infrastructure and foundational team processes and focused less on a individual CVD risk factor care processes. Our study suggests that mental health organizations need administrative and personnel support to conduct quality improvement work, a new direction for many, and an information technology infrastructure to conduct population health for CVD risk factor care among populations with SMI. Caring for populations with SMI requires teams of clinicians within mental and physical health settings. This pilot study offers a novel strategy to deliver complex care coordination for CVD risk factors to a marginalized population with SMI, but much work remains to improve quality improvement culture and self-efficacy for CVD risk factor coordination among mental health providers.

## Data Availability

The raw data supporting the conclusions of this article will be made available by the authors, without undue reservation.
