# Supplementary material for: Pilot of a team-based quality improvement strategy to improve cardiovascular risk factors care in community mental health centers
Source: Front Psychiatry. 2025 Jan 31;16:1446985. doi: 10.3389/fpsyt.2025.1446985 (PMC11825777; doi:10.3389/fpsyt.2025.1446985)
Supplement: Supplementary file 1 [file DataSheet1.docx]

**APPENDIX A: SUPPLEMENTAL TABLES**

### Table A1. Additional pre-implementation characteristics of the staff participants and sites timelines.

| **CHARACTERISTIC** | **ALL SITES**  n (%) | **SITE 1**  n (%) | **SITE 2**  n (%) | **SITE 3**  n (%) | **SITE 4**  n (%) |
| --- | --- | --- | --- | --- | --- |
|  | (n=85) | (n=26) | (n=16) | (n=21) | (n=22) |
| Race |  |  |  |  |  |
| White or Caucasian | 61 (71.8) | 18 (69.2) | 11 (68.8) | 16 (76.2) | 16 (72.7) |
| Black or African-American | 16 (18.8) | 6 (23.1) | 4 (25.0) | 4 (19.0) | 2 (9.1) |
| Asian | 5 (5.9) | 1 (3.8) | 0 (0.0) | 1 (4.8) | 3 (13.6) |
| Native Hawaiian or other Pacific Islander | 1 (1.2) | 1 (3.8) | 0 (0.0) | 0 (0.0) | 0 (0.0) |
| American Indian or Alaska native | 0 (0.0) | 0 (0.0) | 0 (0.0) | 0 (0.0) | 0 (0.0) |
| More than one race^1^ | 2 (2.4) | 0 (0.0) | 1 (6.3) | 0 (0.0) | 1 (4.5) |
| Hispanic/Latino | 6 (7.1) | 2 (7.7) | 2 (12.5) | 1 (4.8) | 1 (4.5) |
| Plan to leave this psychiatric rehabilitation program in the next 12 months? **–** yes or unknown | 13 (15.3) | 4 (15.4) | 4 (25.0) | 2 (9.5) | 3 (13.6) |
| Plan to move in the next 12 months? – yes or unknown | 12 (14.1) | 3 (11.5) | 4 (25.0) | 0 (0.0) | 5 (22.7) |
| No longer employed at organization at 12 months^2^ | 19 (22.4) | 6 (23.1) | 4 (25.0) | 5 (23.8) | 4 (18.2) |
|  |  |  |  |  |  |
| **Site Start Date (duration)** |  |  |  |  |  |
| Pre-Implementation (2 months) |  | 3/12/21 | 7/30/21 | 9/30/21 | 11/18/21 |
| Implementation (12 months) |  |  |  |  |  |
| 0 Months (Baseline) |  | 5/12/21 | 9/30/21 | 11/30/21 | 1/18/22 |
| 6 months |  | 11/12/21 | 3/30/22 | 5/30/22 | 6/18/22 |
| 12 months |  | 5/12/22 | 9/30/22 | 11/30/22 | 1/18/23 |
| Sustainment (3 months) |  | 8/12/22 | 12/30/22 | 2/28/23 | 4/18/23 |

^1^ Those who reported more than one race: 1 Black and white (Site 2) and 1 Black, white, and other (Site 4).

^2^ One was a CUSP team member (Site 3); the others were non-CUSP participants.

### Table A2. Additional pre-implementation characteristics of the health home clients

| **CHARACTERISTIC** | **ALL SITES**  (n=498) | | **SITE 1**  (n=104) | | **SITE 2**  (n=83) | | **SITE 3**  (n=149) | | **SITE 4**  (n=162) | |
| --- | --- | --- | --- | --- | --- | --- | --- | --- | --- | --- |
|  | n^1^ | %^2^ | n^1^ | %^2^ | n^1^ | %^2^ | n^1^ | %^2^ | n^1^ | %^2^ |
| Gender |  |  |  |  |  |  |  |  |  |  |
| Female | 237/445 | 53.3 | 47 | 45.2 | 26 | 31.3 | 81 | 54.4 | 83/109 | 76.1 |
| Male | 206/445 | 46.3 | 56 | 53.8 | 57 | 68.7 | 68 | 45.6 | 25/109 | 22.9 |
| Other | 2/445 | 0.4 | 1 | 1.0 | 0 | 0.0 | 0 | 0.0 | 1/109 | 0.9 |
| Race |  |  |  |  |  |  |  |  |  |  |
| White/Caucasian | 227/442 | 51.4 | 37/98 | 37.8 | 49 | 59.0 | 86 | 57.7 | 55/112 | 50.9 |
| Black/African-American | 200/442 | 45.2 | 59/98 | 60.2 | 30 | 36.1 | 60 | 40.3 | 51/112 | 47.2 |
| Asian | 9/442 | 2.0 | 2/98 | 2.0 | 3 | 3.6 | 3 | 2.0 | 1/112 | 0.9 |
| Native Hawaiian/Other Pacific Islander | 1/442 | 0.2 | 0/98 | 0.0 | 1 | 1.2 | 0 | 0.0 | 0/112 | 0.0 |
| American Indian or Alaska native | 1/442 | 0.2 | 0/98 | 0.0 | 0 | 0.0 | 0 | 0.0 | 1/112 | 0.9 |
| More than one race | 0/442 | 0.0 | 0/98 | 0.0 | 0 | 0.0 | 0 | 0.0 | 0/112 | 0.0 |
| Hispanic/Latino | 13/445 | 2.9 | 5 | 4.8 | 3 | 3.6 | 2 | 1.3 | 3/109 | 2.8 |
| Primary mental health dx |  |  |  |  |  |  |  |  |  |  |
| Bipolar disorder | 112/442 | 25.3 | 18 | 17.3 | 12 | 14.5 | 38 | 25.5 | 44/106 | 41.5 |
| Major depression | 130/442 | 29.4 | 39 | 37.5 | 12 | 14.5 | 37 | 24.8 | 42/106 | 39.6 |
| Schizoaffective disorder | 100/442 | 22.6 | 15 | 14.4 | 23 | 27.7 | 49 | 32.9 | 13/106 | 12.3 |
| Schizophrenia | 90/442 | 20.4 | 29 | 27.9 | 33 | 39.8 | 22 | 14.8 | 6/106 | 5.7 |
| Other psychotic disorder | 10/442 | 2.3 | 3 | 2.9 | 3 | 3.6 | 3 | 2.0 | 1/106 | 0.9 |
| Receiving disability | 297/436 | 68.1 | 31 | 29.8 | 42 | 50.6 | 148 | 99.3 | 76/100 | 76.0 |
| History of alcohol or substance use disorder | 146/440 | 33.2 | 29 | 27.9 | 38 | 45.8 | 35 | 23.5 | 44/104 | 42.3 |
| Age – mean (SD) | n=444 | 47.5 (14.2) | n=104 | 44.1 (15.2) | n=83 | 49.7 (14.0) | n=149 | 48.3 (14.5) | n=108 | 48.1 (12.2) |
| BMI – mean (SD) | n=402 | 33.9 (9.2) | n=104 | 32.3 (8.6) | n=79 | 33.8 (8.2) | n=122 | 33.9 (9.1) | n=97 | 35.8 (10.5) |
| Number of CVD risk factors |  |  |  |  |  |  |  |  |  |  |
| 0 | 31/439 | 7.1 | 5 | 4.8 | 5 | 6.0 | 15 | 10.1 | 6/103 | 5.8 |
| 1 | 112/439 | 25.5 | 30 | 28,8 | 16 | 19.3 | 45 | 30.2 | 21/103 | 20.4 |
| 2 | 109/439 | 24.8 | 29 | 27.9 | 21 | 25.3 | 38 | 25.5 | 21/103 | 20.4 |
| 3 | 100/439 | 22.8 | 19 | 18.3 | 17 | 20.5 | 28 | 18.8 | 36/103 | 35.0 |
| 4 | 65/439 | 14.8 | 11 | 10.6 | 16 | 19.3 | 23 | 15.4 | 15/103 | 14.6 |
| 5 | 22/439 | 27.8 | 10 | 9.6 | 8 | 9.6 | 0 | 0.0 | 4/103 | 3.9 |

^1^ Denominators given if data is unavailable for a specific item.

^2^ % unless otherwise indicated as mean (SD)

**Table A3.** Summary of by site changes across the 12-month implementation stage (pre-implementation to post-implementation) for primary outcomes: quality improvement culture and perceived provider self-efficacy for cardiovascular disease risk factor coordination.

|  | **Site 1** | | | | **Site 2** | | | | **Site 3** | | | | **Site 4** | | | |
| --- | --- | --- | --- | --- | --- | --- | --- | --- | --- | --- | --- | --- | --- | --- | --- | --- |
|  | Pre | Post | Change | | Pre | Post | Change | | Pre | Post | Change | | Pre | Post | Change | |
|  | M (SD)  [n] | M (SD)  [n] | Est. (95% CI) ^3^ | d ^4^ | M (SD)  [n] | M (SD)  [n] | Est. (95% CI) ^3^ | d ^4^ | M (SD)  [n] | M (SD)  [n] | Est. (95%CI) ^3^ | d ^4^ | M (SD)  [n] | M (SD)  [n] | Est. (95% CI) ^3^ | d ^4^ |
| **Quality Improvement Culture^1^** | | | | | | | | | | | | | | | | |
| All participants | 3.5 (0.9) [25] | 3.5 (0.7) [19] | 0.0  (-0.2, 0.2) | 0.03 | 3.4 (0.6)  [16] | 3.4 (0.5) [11] | 0.0  (-0.3, 0.3) | 0.02 | 4.0 (0.4) [21] | 4.0 (0.4) [16] | 0.0  (-0.3, 0.2) | -0.06 | 4.2 (0.4) [22] | 4.1 (0.5) [18] | -0.1  (-0.4, 0.1) | -0.20 |
| Non-CUSP only | 3.6 (0.8) [18] | 3.5 (0.6) [13] | 0.0  (-0.2, 0.2) | 0.03 | 3.3 (0.6) [9] | 3.5 (0.6) [5] | 0.0  (-0.3, 0.3) | 0.02 | 4.0 (0.5) [17] | 4.0 (0.4) [13] | 0.0  (-0.3, 0.2) | -0.06 | 4.3 (0.4) [18] | 4.1 (0.4) [14] | -0.1  (-0.4, 0.1) | -0.20 |
| CUSP Team | 3.0 (1.0) [7] | 3.3 (1.0) [6] | 0.0  (-0.2, 0.2) | 0.03 | 3.4 (0.8) [7] | 3.4 (0.5) [6] | 0.0  (-0.3, 0.3) | 0.02 | 4.1 (0.4) [4] | 3.6 (0.3) [3] | 0.0  (-0.3, 0.2) | -0.06 | 4.1 (0.6) [4] | 4.1 (0.7) [4] | 0.01  (-0.4, 0.1) | -0.20 |
| **Self-Efficacy to Coordinate Care^2^** | | | | | | | | | | | | | | | | |
| Hypertension | 5.3 (2.6)  [7] | 7.4 (1.1)  [6] | 2.1 (0.7, 3.5) * | 0.94 | 6.8 (1.2)  [7] | 6.8 (1.1)  [6] | 0.1 (-1.5 , 1.3) | -0.04 | 4.1 (1.6)  [4] | 6.4 (0.9)  [3] | 2.1  (0.2, 4.1) * | 0.94 | 7.7 (2.3)  [4] | 7.5 (1.9)  [4] | -0.2 (-2.0, 1.6) | -0.07 |
| Dyslipidemia | 4.8 (2.6)  [7] | 7.0 (1.3)  [6] | 2.1 (0.3, 3.9) * | 0.89 | 7.1 (1.1)  [7] | 7.2 (1.4)  [6] | 0.0 (-1.8 , 1.9) | 0.02 | 3.8 (1.9)  [4] | 6.4 (0.9)  [3] | 2.6  (0.1, 5.0) * | 1.07 | 7.6 (2.0)  [4] | 7.1 (2.0)  [4] | -0.5 (-2.8, 1.8) | -0.21 |
| Diabetes | 5.5 (2.6)  [7] | 7.0 (1.1)  [6] | 1.6 (-0.1 , 3.2) | 0.61 | 7.4 (2.2)  [7] | 7.6 (1.4)  [6] | 0.0 (-1.7 , 1.6) | -0.02 | 4.3 (2.8)  [4] | 6.7 (2.3)  [3] | 2.3  (0.0, 4.5) * | 0.88 | 7.3 (2.3)  [4] | 7.7 (1.6)  [4] | 0.4 (-1.8, 2.5) | 0.15 |

*p<0.05

^1^ Quality Improvement Culture scale: 1 (needs improvement) to 5 (strong culture). Completed by all staff and CUSP team members.

^2^ Health Home Self-Efficacy scale: 0 (no self-efficacy) to 10 (high self-efficacy). Completed by CUSP team members.

^3^ Estimates derived from outcome specific mixed-effects repeated measures regression models utilizing all available data with fixed effects for study site, CUSP team status, and a visit by site interaction for the Quality Improvement Culture. For the Self-Efficacy the model is the same but without fixed effects for CUSP team status as all respondents are a part of the CUSP team.

^4^ Cohen’s d with interpretation 0.00-0.19 = Very Small, 0.20-0.49 = Small, 0.50-0.79 = Medium, 0.80+ = Large from Cohen d statistic.

**Table A4**. Summary of changes across the 12-month implementation stage (pre-implementation to post-implementation) for quality improvement culture survey sub-scores.

|  | **Pre** | | **Post** | | **Change** | | |
| --- | --- | --- | --- | --- | --- | --- | --- |
|  | n | M (SD) | n | M (SD) | Estimate (95% CI) ^2^ | Cohen’s d | Effect size ^3^ |
| **Quality Improvement Culture^1^** | | | | | | | |
| 1. Teamwork Within Teams | 84 | 4.1 (0.9) | 64 | 4.0 (0.8) | -0.1 (-0.2, 0.1) | -0.06 | Very Small |
| 2. Supervisor/Manager Expectations & Actions Promoting Quality Improvement | 84 | 4.0 (0.9) | 64 | 4.1 (0.8) | 0.0 (-0.2, 0.2) | -0.01 | Very Small |
| 3. Organizational Learning - Continuous Improvement | 84 | 3.9 (0.8) | 64 | 3.9 (0.6) | 0.0 (-0.2, 0.1) | -0.04 | Very Small |
| 4. Management Support for Patient Safety | 84 | 3.7 (1.0) | 64 | 3.7 (0.8) | 0.0 (-0.2, 0.2) | -0.05 | Very Small |
| 5. Overall Perceptions of Quality Improvement | 84 | 3.7 (0.9) | 64 | 3.6 (0.8) | -0.1 (-0.3, 0.1) | -0.16 | Very Small |
| 6. Feedback & Communication About Error | 84 | 3.8 (0.9) | 64 | 3.7 (0.8) | -0.1 (-0.3, 0.0) | -0.17 | Very Small |
| 7. Communication Openness | 84 | 3.5 (0.8) | 64 | 3.5 (0.8) | 0.0 (-0.1, 0.2) | 0.05 | Very Small |
| 8. Frequency of Events Reported | 84 | 4.0 (0.9) | 64 | 4.1 (0.8) | 0.1 (-0.1, 0.3) | 0.09 | Very Small |
| 9. Teamwork Across Teams | 84 | 3.9 (0.9) | 64 | 3.9 (0.8) | 0.0 (-0.2, 0.2) | <0.01 | Very Small |
| 10. Staffing | 84 | 2.9 (0.9) | 64 | 2.8 (0.9) | -0.2 (-0.4, 0.0) | -0.19 | Very Small |

*p<0.05

^1^ Quality Improvement Culture scale: 1 (needs improvement) to 5 (strong culture). Completed by all staff and CUSP team members.

^2^ Estimates derived from outcome specific mixed-effects repeated measures regression models utilizing all available data with fixed effects for study site and CUSP team status.

^3^ Effect size interpretation from Cohen d statistic.

**Table A5**. Summary of changes by site across the 12-month implementation stage (pre-implementation to post-implementation) for quality improvement culture survey sub-scores.

|  | **Site 1** | | | | **Site 2** | | | | **Site 3** | | | | **Site 4** | | | |
| --- | --- | --- | --- | --- | --- | --- | --- | --- | --- | --- | --- | --- | --- | --- | --- | --- |
|  | Pre | Post | Change | | Pre | Post | Change | | Pre | Post | Change | | Pre | Post | Change | |
|  | M (SD) | M (SD) | Est. (95% CI) ^2^ | d ^3^ | M (SD) | M (SD) | Est. (95% CI) ^2^ | d ^3^ | M (SD) | M (SD) | Est. (95%CI) ^2^ | d ^3^ | M (SD) | M (SD) | Est. (95% CI) ^2^ | d ^3^ |
| **Quality Improvement Culture^1^** | | | | | | | | | | | | | | | | |
|  | *n=25* | *n=19* |  |  | *n=16* | *n=11* |  |  | *n=21* | *n=16* |  |  | *n=22* | *n=16* |  |  |
| 1. Teamwork Within Teams | 3.7 (1.1) | 3.5 (1.0) | -0.1  (-0.4, 0.1) | -0.16 | 3.7 (0.8) | 3.7 (0.6) | 0.0  (-0.4, 0.3) | -0.04 | 4.4 (0.7) | 4.4 (0.5) | -0.1  (-0.4, 0.2) | -0.07 | 4.5 (0.5) | 4.6 (0.5) | 0.0  (-0.2, 0.3) | 0.04 |
| 2. Supervisor/ Manager Expectations & Actions Promoting Quality Improvement | 3.6 (1.1) | 4.0 (0.8) | 0.3 (0.0, 0.7) | 0.36 | 3.8 (0.9) | 3.8 (0.9) | 0.0  (-0.4, 0.4) | 0.00 | 4.1 (0.5) | 4.1 (0.6) | -0.1  (-0.4, 0.3) | -0.06 | 4.6 (0.4) | 4.2 (0.7) | -0.3 (-0.7, 0.0) | -0.38 |
| 3. Organizational Learning - Continuous Improvement | 3.6 (1.0) | 3.7 (0.8) | 0.1  (-0.2, 0.4) | 0.08 | 3.6 (0.8) | 3.7 (0.8) | 0.0  (-0.4, 0.4) | -0.01 | 4.1 (0.5) | 4.1 (0.3) | 0.0  (-0.3, 0.4) | 0.05 | 4.2 (0.4) | 4.0 (0.5) | -0.2  (-0.5, 0.1) | -0.28 |
| 4. Management Support for Patient Safety | 3.4 (1.1) | 3.3 (1.0) | -0.1  (-0.4, 0.3) | -0.05 | 3.2 (0.9) | 3.5 (0.8) | 0.2  (-0.3, 0.7) | 0.17 | 4.1 (0.6) | 4.0 (0.6) | 0.0  (-0.4, 0.4) | -0.03 | 4.2 (0.8) | 4.0 (0.8) | -0.2  (-0.6, 0.2) | -0.18 |
| 5. Overall Perceptions of Quality Improvement | 3.4 (0.9) | 3.3 (0.9) | 0.0  (-0.4, 0.3) | -0.06 | 3.3 (0.9) | 3.1 (0.7) | -0.3  (-0.7, 0.2) | -0.32 | 3.9 (0.6) | 3.9 (0.5) | 0.0  (-0.4, 0.3) | -0.06 | 4.1 (0.7) | 3.9 (0.8) | -0.2  (-0.6, 0.1) | -0.25 |
| 6. Feedback & Communication About Error | 3.5 (1.0) | 3.5 (0.9) | 0.1  (-0.2, 0.4) | 0.11 | 3.5 (0.9) | 3.4 (0.8) | -0.2  (-0.6, 0.2) | -0.23 | 4.1 (0.5) | 4.0 (0.6) | -0.2  (-0.6, 0.1) | -0.24 | 4.2 (0.7) | 3.8 (0.9) | -0.3  (-0.6, 0.0) | -0.37 |
| 7. Communication Openness | 3.2 (0.8) | 3.0 (0.9) | -0.2  (-0.5, 0.2) | -0.19 | 3.1 (0.8) | 3.6 (0.5) | 0.5 (0.1, 0.9) | 0.58 | 3.6 (0.7) | 3.8 (0.7) | 0.2  (-0.2, 0.5) | 0.22 | 4.0 (0.7) | 3.8 (0.7) | -0.2  (-0.5, 0.2) | -0.20 |
| 8. Frequency of Events Reported | 3.7 (1.0) | 3.9 (1.1) | 0.2  (-0.1, 0.5) | 0.25 | 4.0 (0.9) | 3.9 (0.9) | -0.1  (-0.5, .4) | -0.07 | 4.2 (0.8) | 4.0 (0.5) | -0.1  (-0.5, 0.3) | -0.11 | 4.2 (0.7) | 4.4 (0.7) | 0.2  (-0.2, 0.5) | 0.21 |
| 9. Teamwork Across Teams | 3.6 (1.0) | 3.6 (0.9) | 0.0  (-0.3, 0.3) | 0.00 | 3.2 (0.7) | 3.4 (0.6) | 0.1  (-0.3, 0.6) | 0.16 | 4.1 (0.8) | 4.1 (0.6) | 0.0  (-0.3, 0.4) | 0.02 | 4.4 (0.6) | 4.3 (0.5) | -0.1  (-0.4, 0.2) | -0.13 |
| 10. Staffing | 3.1 (0.9) | 3.0 (0.8) | -0.2  (-0.5, 0.2) | -0.18 | 2.0 (0.7) | 1.8 (0.9) | -0.2  (-0.7, 0.3) | -0.25 | 2.7 (0.8) | 2.7 (0.6) | 0.2  (-0.4, 0.4) | -0.04 | 3.5 (0.7) | 3.2 (0.8) | -0.3  (-0.7, 0.1) | -0.30 |

*p<0.05

^1^ Quality Improvement Culture scale: 1 (needs improvement) to 5 (strong culture). Completed by all staff and CUSP team members.

^2^ Estimates derived from outcome specific mixed-effects repeated measures regression models utilizing all available data with fixed effects for study site, CUSP team status, and a visit by site interaction.

^3^ Cohen’s d with interpretation 0.00-0.19 = Very Small, 0.20-0.49 = Small, 0.50-0.79 = Medium, 0.80+ = Large from Cohen d statistic.

**Table A6.** Summary of by site outcomes for acceptability, appropriateness, and feasibility of the CUSP implementation strategy and evidence bundle to improve delivery of cardiovascular risk factor care coordination.

|  | **Site 1** | | | | **Site 2** | | | | **Site 3** | | | | **Site 4** | | | |
| --- | --- | --- | --- | --- | --- | --- | --- | --- | --- | --- | --- | --- | --- | --- | --- | --- |
|  | Pre | Post | Change | | Pre | Post | Change | | Pre | Post | Change | | Pre | Post | Change | |
|  | M (SD) | M (SD) | Est. (95% CI) ^2^ | d^3^ | M (SD) | M (SD) | Est. (95% CI) ^2^ | d^3^ | M (SD) | M (SD) | Est. (95%CI) ^2^ | d^3^ | M (SD)  [n] | M (SD)  [n] | Est. (95% CI) ^2^ | d^3^ |
| **Acceptability, Appropriateness, and Feasibility^1^** | | | | | | | | | | | | | | | | |
| *CUSP Strategy* | *n=6* | *n=6* |  |  | *n=5* | *n=6* |  |  | *n=4* | *n=3* |  |  | *n=4* | *n=4* |  |  |
| Acceptability | 4.4 (0.6) | 4.6 (0.5) | 0.2  (-0.4, 0.8) | 0.36 | 4.4 (0.5) | 3.9 (0.3) | -0.5  (-1.1, 0.2) | -0.98 | 4.4 (0.5) | 3.8 (0.4) | -0.6  (-1.4, 0.2) | -1.27 | 4.3 (0.3) | 4.3 (0.9) | 0.0  (-0.8, 0.7) | -0.07 |
| Appropriateness | 4.5 (0.5) | 4.6 (0.5) | 0.1  (-0.6, 0.8) | 0.13 | 4.4 (0.5) | 3.9 (0.6) | -0.5  (-1.2, 0.2) | -1.16 | 4.3 (0.5) | 3.9 (0.2) | -0.4  (-1.3, 0.5) | -0.87 | 4.3 (0.2) | 4.2 (1.0) | -0.1 (1.0 , 0.7) | -0.28 |
| Feasibility | 4.3 (0.4) | 4.6 (0.5) | 0.3  (-0.3, 0.9) | 0.76 | 4.3 (0.5) | 3.9 (0.4) | -0.5  (-1.1, 0.1) | -1.22 | 4.3 (0.5) | 3.8 (0.3) | -0.5  (-1.2, 0.3) | -1.17 | 4.2 (0.2) | 4.3 (1.0) | 0.1  (-0.6, 0.8) | 0.24 |
| *Evidence Bundle* | *n=6* | *n=6* |  |  | *n=5* | *n=6* |  |  | *n=4* | *n=3* |  |  | *n=4* | *n=4* |  |  |
| Acceptability | 4.5 (0.6) | 4.5 (0.5) | 0.0  (-0.6, 0.6) | 0.00 | 4.2 (0.4) | 4.0 (0.3) | -0.2  (-0.8, 0.3) | -0.49 | 4.8 (0.5) | 3.8 (0.4) | -0.9  (-1.6, -0.2) * | -1.92 | 4.4 (0.4) | 4.4 (0.7) | 0.0  (-0.7, 0.7) | 0.06 |
| Appropriateness | 4.5 (0.5) | 4.6 (0.5) | 0.1  (-0.5, 0.6) | 0.13 | 4.2 (0.4) | 4.0 (0.5) | -0.2  (-0.8, 0.3) | -0.47 | 4.6 (0.5) | 3.8 (0.4) | -0.7  (-1.5, 0.0) * | -0.77 | 4.0 (0.2) | 4.2 (1.1) | 0.2  (-0.5, 0.8) | 0.63 |
| Feasibility | 4.3 (0.4) | 4.6 (0.5) | 0.2  (-0.3, 0.8) | 0.53 | 4.2 (0.5) | 3.9 (0.4) | -0.3  (-0.8, 0.3) | -0.66 | 4.6 (0.5) | 3.8 (0.3) | -0.7  (-1.5, 0.0) * | -1.75 | 4.2 (0.2) | 4.3 (1.0) | 0.1  (-0.6, 0.7) | 0.22 |

*p<0.05

^1^ Acceptability, Appropriateness, and Feasibility scale: 1 (not acceptable/appropriate/feasible) to 5 (highly acceptable/appropriate/feasible). Completed by CUSP Team.

^2^ Estimates derived from outcome specific mixed-effects regression models utilizing all available data, adjusting for study site, and a visit by site interaction.

^3^ Cohen’s d with interpretation 0.00-0.19 = Very Small, 0.20-0.49 = Small, 0.50-0.79 = Medium, 0.80+ = Large from Cohen d statistics.

**Table A7.** Number and percentage of clients by site with hypertension, diabetes, and dyslipidemia with available clinical data and those who have reached the recommended target for blood pressure control, glycemic control as measured by hemoglobin A1c, and cholesterol control across the 12-month implementation stage (pre-implementation vs. post-implementation).

|  | **Pre** | **Post** | **Pre** | **Post** | **Change** | **Pre** | **Post** | **Change** | **Pre** | **Post** | **Change** |
| --- | --- | --- | --- | --- | --- | --- | --- | --- | --- | --- | --- |
|  | **n** | **n** | **n (%)** | **n (%)** | **OR**  **(95% CI) ^1^** | **n (%)** | **n (%)** | **OR**  **(95% CI) ^1^** | **n (%)** | **n (%)** | **OR**  **(95% CI) ^1^** |
| **Hypertension** | **Identified ^5^** | | **Available BP Data** | | | **BP < 130/80 ^2^** | | | **BP within 1 year** | | |
| All Sites | 189 | 203 | 166 (88) | 186 (92) | 1.8 (1.0, 3.3) | 41 (25) | 59 (32) | 1.4 (0.9, 2.2) | 156 (94) | 168 (90) | 0.7 (0.3, 1.5) |
| Site 1 | 46 | 41 | 46 (100) | 41 (100) | - ^6^ | 10 (22) | 14 (35) | 2.1 (0.8, 5.2) | 41 (89) | 40 (100) | - ^6^ |
| Site 2 | 43 | 39 | 39 (91) | 37 (95) | 1.8 (0.4, 7.4) | 12 (31) | 10 (27) | 0.8 (0.3, 2.2) | 38 (97) | 30 (81) | 0.1 (0.0, 1.0) |
| Site 3 | 50 | 53 | 37 (74) | 47 (89) | 3.2 (1.4, 7.5) | 10 (27) | 16 (34) | 1.4 (0.6, 3.3) | 35 (95) | 44 (94) | 0.9 (0.1, 5.7) |
| Site 4 | 50 | 70 | 44 (88) | 61 (87) | 0.9 (0.3, 2.8) | 9 (21) | 19 (31) | 1.7 (0.8, 3.6) | 42 (96) | 54 (89) | 0.4 (0.1, 1.9) |
| Multi-Site |  |  | 120 (84) | 145 (90) | 1.8 (0.9, 3.4) | 41 (25) | 59 (32) | 1.4 (0.9, 2.2) | 115 (96) | 128 (88) | 0.3 (0.1, 1.0) |
| **Diabetes** | **Identified ^5^** | | **Available Lab Data** | | | **HgbA1c < 7% ^3^** | | | **HgbA1c within 1 year** | | |
| All Sites | 123 | 120 | 92 (75) | 99 (83) | 1.8 (1.2, 2.6) | 53 (58) | 55 (56) | 1.0 (0.7,1.4) | 81 (88) | 83 (84) | 0.3 (0.1, 0.6) |
| Site 1 | 24 | 21 | 23 (96) | 21 (100) | - ^6^ | 10 (44) | 11 (52) | 1.5 (0.6, 3.6) | 17 (71) | 21 (100) | - ^6^ |
| Site 2 | 29 | 26 | 15 (52) | 16 (62) | 1.4 (0.9, 2.2) | 12 (80) | 12 (75) | 1.2 (0.5, 2.7) | 13 (87) | 13 (81) | 0.3 (0.0, 2.6) |
| Site 3 | 34 | 33 | 23 (68) | 27 (82) | 2.2 (1.1, 4.4) | 14 (61) | 14 (52) | 0.7 (0.4, 1.2) | 20 (87) | 22 (82) | 0.7 (0.2, 2.8) |
| Site 4 | 36 | 40 | 31 (86) | 35 (88) | 1.8 (0.7, 4.4) | 17 (55) | 18 (51) | 0.9 (0.5, 1.8) | 31 (100) | 27 (77) | - ^6^ |
| Multi-Site |  |  | 69 (70) | 78 (79) | 1.8 (1.2, 2.7) | 53 (58) | 55 (56) | 1.0 (0.7, 1.5) | 33 (87) | 35 (81) | 0.5 (0.1, 1.7) |
| **Dyslipidemia** | **Identified ^5^** | | **Available Lab Data** | | | **LDL-C < 100 mg/dl ^4^** | | | **Lipids within 1 year** | | |
| All Sites | 166 | 184 | 131 (79) | 133 (72) | 1.2 (1.0, 1.6) | 70 (53) | 75 (56) | 1.4 (1.1, 1.9) | 108 (82) | 78 (59) | 0.3 (0.2, 0.6) |
| Site 1 | 38 | 31 | 38 (100) | 31 (100) | - ^6^ | 22 (58) | 22 (71) | 1.8 (1.1, 2.9) | 26 (68) | 20 (67) | 1.0 (0.4, 2.4) |
| Site 2 | 34 | 32 | 18 (53) | 17 (53) | 1.0 (0.9, 1.1) | 11 (61) | 11 (65) | 1.2 (0.7, 2.1) | 17 (94) | 4 (24) | 0.0 (0.0, 0.3) |
| Site 3 | 53 | 56 | 47 (89) | 49 (88) | 1.1 (0.7, 1.7) | 27 (57) | 29 (59) | 1.1 (0.8, 1.7) | 37 (79) | 32 (65) | 0.5 (0.2, 1.2) |
| Site 4 | 41 | 65 | 28 (68) | 36 (55) | 1.6 (1.0, 2.7) | 10 (24) | 13 (36) | 2.0 (0.8, 4.6) | 28 (100) | 22 (61) | - ^6^ |
| Multi-Site |  |  | 93 (73) | 102 (67) | 1.2 (1.0, 1.5) | 70 (53) | 75 (56) | 1.5 (1.1, 2.0) | 80 (64) | 56 (47) | 0.2 (0.1, 0.6) |

Blood pressure (BP); Hemoglobin A1c (HgbA1c); Low density lipoprotein cholesterol (LDL-C).

^1^ All Sites odds ratio was derived from binomial generalized estimating equations (GEE) models utilizing all available data from all four study sites and adjusting for study site. Site specific and multi-site odds ratio was each derived from binomial generalized estimating equations (GEE) models utilizing all available data from included sites, with study site main effect for included sites, and corresponding visit by site interaction effects for included sites. An excluded site was one that had a homogeneous outcome responses (e.g., 100%) at one or both time points so that a numerical solution cannot be derived through modeling. It is noted for each outcome where a site could not be included within this modeling.

^2^ Strict control for blood pressure is defined as <130/80 mmHg per national guidelines from the American College of Cardiology/American Heart Association.

^3^ Strict glycemic control is defined as HgbA1c <7% by the American Diabetes Association.

^4^ Strict cholesterol control is defined as LDL-C <100 mg/dl by the American College of Cardiology/American Heart Association.

^5^ Clients identified by health home nurse as having the given cardiovascular disease (CVD) risk factor. Differences in subpopulation size with a given CVD risk factor reflect clients enrolling and disenrolling at participating sites and sites/primary care identifying new medical conditions. Only clients with the CVD risk factor were included for each CVD risk factor.

^6^ Site could not be included in the multi-site modeling due to the small sample size and homogeneity of responses.

**Table A8.** Number and percentage of clients by site with hypertension, diabetes, and dyslipidemia with available clinical data and those who have reached the recommended lenient control targets for blood pressure control, glycemic control as measured by hemoglobin A1c, and cholesterol control across the 12-month implementation stage (pre-implementation vs. post-implementation).

|  | **Pre** | **Post** | **Pre** | **Post** | **Pre** | **Post** | **Change** |
| --- | --- | --- | --- | --- | --- | --- | --- |
|  | **n** | **n** | **n (%)** | **n (%)** | **n (%)** | **n (%)** | **OR**  **(95% CI) ^1^** |
| **Hypertension** | **Identified ^5^** | | **Available BP Data** | | **BP < 140/90 ^2^** | | |
| All Sites | 189 | 203 | 166 (88) | 186 (92) | 94 (57) | 119 (64) | 1.4 (1.0, 2.0) |
| Site 1 | 46 | 41 | 46 (100) | 41 (100) | 26 (57) | 33 (83) | 3.0 (1.2, 7.8) |
| Site 2 | 43 | 39 | 39 (91) | 37 (95) | 26 (67) | 26 (70) | 1.0 (0.5, 2.2) |
| Site 3 | 50 | 53 | 37 (74) | 47 (89) | 21 (57) | 24 (51) | 0.8 (0.4, 1.8) |
| Site 4 | 50 | 70 | 44 (88) | 61 (87) | 21 (48) | 36 (59) | 1.5 (0.8, 2.8) |
| Multi-Site |  |  |  |  | 94 (57) | 119 (64) | 1.4 (0.9, 2.1) |
| **Diabetes** | **Identified ^5^** | | **Available Lab Data** | | **HgbA1c < 8% ^3^** | | |
| All Sites | 123 | 120 | 92 (75) | 99 (83) | 63 (69) | 70 (71) | 1.1 (0.7, 1.7) |
| Site 1 | 24 | 21 | 23 (96) | 21 (100) | 15 (65) | 16 (71) | 1.3 (0.4, 4.0) |
| Site 2 | 29 | 26 | 15 (52) | 16 (62) | 13 (87) | 15 (94) | 2.4 (0.4, 14.8) |
| Site 3 | 34 | 33 | 23 (68) | 27 (82) | 16 (70) | 19 (70) | 0.8 (0.4, 1.5) |
| Site 4 | 36 | 40 | 31 (86) | 35 (88) | 19 (61) | 21 (60) | 1.0 (0.5, 1.9) |
| Multi-Site |  |  |  |  | 63 (69) | 70 (71) | 1.2 (0.7, 2.2) |
| **Dyslipidemia** | **Identified ^5^** | | **Available Lab Data** | | **LDL-C < 130 mg/dl ^4^** | | |
| All Sites | 166 | 184 | 131 (79) | 133 (72) | 100 (76) | 98 (74) | 0.9 (0.6, 1.4) |
| Site 1 | 38 | 31 | 38 (100) | 31 (100) | 29 (76) | 27 (87) | 1.6 (0.8, 3.1) |
| Site 2 | 34 | 32 | 18 (53) | 17 (53) | 15 (83) | 15 (88) | 0.7 (0.1, 5.2) |
| Site 3 | 53 | 56 | 47 (89) | 49 (88) | 38 (81) | 36 (74) | 0.7 (0.3, 1.6) |
| Site 4 | 41 | 65 | 28 (68) | 36 (55) | 18 (64) | 20 (56) | 0.9 (0.4, 1.7) |
| Multi-Site |  |  |  |  | 100 (76) | 98 (74) | 0.9 (0.5, 1.7) |

Blood pressure (BP); Hemoglobin A1c (HgbA1c); Low density lipoprotein cholesterol (LDL-C).

^1^ All Sites odds ratio was derived from binomial generalized estimating equations (GEE) models utilizing all available data from all four study sites and adjusting for study site. Site specific and multi-site odds ratio was each derived from binomial generalized estimating equations (GEE) models utilizing all available data from included sites, with study site main effect for included sites, and corresponding visit by site interaction effects for included sites. An excluded site was one that had a homogeneous outcome responses (e.g., 100%) at one or both time points so that a numerical solution cannot be derived through modeling. It is noted for each outcome where a site could not be included within this modeling.

^2^ Lenient control for blood pressure is defined as <140/90 mmHg per national guidelines from the American College of Cardiology/American Heart Association.

^3^ Lenient glycemic control is defined as HgbA1c <8% by the American Diabetes Association.

^4^ Lenient cholesterol control is defined as LDL-C <100 mg/dl by the American College of Cardiology/American Heart Association.

^5^ Clients identified by health home nurse as having the given cardiovascular disease (CVD) risk factor. Differences in subpopulation size with a given CVD risk factor reflect clients enrolling and disenrolling at participating sites and sites/primary care identifying new medical conditions. Only clients with the CVD risk factor were included for each CVD risk factor.

Appendix B: Evidence Bundle Table of Contents

[Overview of the Implementation Guide: 14](#_Toc120616629)

[Part I: Clinical Guidelines Bundle for Cardiovascular Risk Factors for People with Serious Mental Illness 15](#_Toc120616630)

[Overview: Using the Clinical Guidelines Bundle: 15](#_Toc120616631)

[Clinical Considerations Across All Cardiovascular Disease (CVD) Risk Factors 16](#_Toc120616632)

[Dietary and Lifestyle Modifications 16](#_Toc120616633)

[ASCVD Risk Estimator 16](#_Toc120616634)

[Aspirin use 16](#_Toc120616635)

[Metabolic syndrome 16](#_Toc120616636)

[Smoking cessation 16](#_Toc120616637)

[DIABETES MELLITUS 17](#_Toc120616638)

[Guidelines 17](#_Toc120616639)

[Clinical Considerations for People with Serious Mental Illness 18](#_Toc120616640)

[Supplemental Medical Information 18](#_Toc120616641)

[Common Medications for Diabetes Mellitus Management 20](#_Toc120616642)

[ELEVATED BLOOD CHOLESTEROL (DYSLIPIDEMIA) 21](#_Toc120616643)

[Guidelines 21](#_Toc120616644)

[Clinical Considerations for People with Serious Mental Illness 23](#_Toc120616645)

[Supplemental Medical Information 23](#_Toc120616646)

[Common Medications for Lipid Management 24](#_Toc120616647)

[HYPERTENSION 25](#_Toc120616648)

[Guidelines 25](#_Toc120616649)

[Clinical Considerations for People with Serious Mental Illness 26](#_Toc120616650)

[Supplemental Medical Information 26](#_Toc120616651)

[Common Medications 27](#_Toc120616652)

[PART II: Cross-Cutting Care Processes to Deliver CVD Risk Factor Care for People with Serious Mental Illness 28](#_Toc120616653)

[POPULATION HEALTH MANAGEMENT 28](#_Toc120616654)

[Overview 28](#_Toc120616655)

[Identify and Track CVD risk factors 28](#_Toc120616656)

[Plan to Improve Specific CVD Risk Factor(s) 28](#_Toc120616657)

[Tips for Using the Population Health Database 29](#_Toc120616658)

[INDIVIDUAL CLIENT CARE PLAN 31](#_Toc120616659)

[Overview 31](#_Toc120616660)

[Create an Individualized *Care Plan* 31](#_Toc120616661)

[Use the Care Plan to Guide Actions 31](#_Toc120616662)

[Provide Supportive Self-management 31](#_Toc120616663)

[CARE COORDINATION 31](#_Toc120616664)

[Overview 32](#_Toc120616665)

[Prior to PCP Visit: 32](#_Toc120616666)

[During PCP Visit: 33](#_Toc120616667)

[After/in between PCP visit: 33](#_Toc120616668)

[Post-Hospital Discharge 34](#_Toc120616669)

[Additional Tools/Handouts 34](#_Toc120616670)

[Educational handouts 35](#_Toc120616671)

[Appointment Reminder 44](#_Toc120616672)

[Chronic Disease Logs: 45](#_Toc120616673)

[Blood Glucose Log 45](#_Toc120616674)

[Blood Pressure Log 46](#_Toc120616675)

[Lifestyle Log: Diet and Exercise Tracker 48](#_Toc120616676)

[Medication Card for client 49](#_Toc120616677)

[Primary Care Physician Phone Call Log 50](#_Toc120616678)

[Primary Care Visit Communication Form 51](#_Toc120616679)

[Guides for Effective Communication 52](#_Toc120616680)

[References 59](#_Toc120616681)

# Overview of Evidence Bundle:

In this evidence bundle and implementation guide, we provide a) summary of clinical guidelines for the diagnosis and management of diabetes, elevated blood cholesterol, and hypertension; b) cross-cutting steps on conducting population health management, care management, and care coordination using evidence-based tea practices to direct your strategies and priorities. We also introduce a package of tools that can be used to manage the cardiovascular-related health needs of health home clients. These tools may be adapted for use in your own health home. This guide is structured around 3 concepts: population health management, the individual client care plan, and care coordination.

The three concepts are interconnected. Using the provided population health management tool (e.g. the Population Health Database) will help your CUSP team identify the CVD risk factor(s) affecting health home clients. Updating this database over time will help you track client progress. The information on the Individual client care plan will feed into the Population Health Management Database and will inform what care coordination steps are needed to effect improvements in cardiovascular risk factors for individual clients and the health home population as a whole.

# Part I: Clinical Guidelines Bundle for Cardiovascular Risk Factors for People with Serious Mental Illness

| **Key Documents and Tools in this Section**   - Clinical Guidelines Bundle   **Other Documents and Tools Referenced in this Section** |
| --- |
| - Care Plan (*Individual Client Care Plan* section) |

## Overview: Using the Clinical Guidelines Bundle:

In this section, we summarize the clinical management around three common cardiovascular disease risk factors: diabetes, dyslipidemia, and hypertension. We have assembled a set of recommendations for dietary and lifestyle recommendations and clinical considerations that are applicable to all cardiovascular risk factors.

For each risk factor, you will find information on a) guidelines, b) clinical considerations for clients with serious mental illness, c) supplemental information, and d) common medications. It is our hope that these guidelines will help Health Home teams learn best practices for each cardiovascular risk factor care. Please use this information to support the population health, individual care plan, and care coordination activities of your Health Home. Using these guidelines can help teams and clients become effective partners with clients’ primary care providers in the management of cardiovascular risk factors.

1. Identify the cardiovascular risk factors of interest to a specific client or clients.
   1. The ***Population Health Database*** should be color-coded to visually identify risk factors that are not well controlled.
   2. At the client level, these guidelines will help direct the ***Care Plan*** and necessary care coordination activities.
2. Use the clinical guidelines section to identify the necessary action items in tandem with individual care plan and care coordination activities.
   1. Find the guideline of interest with associated information (e.g. screening, management, testing, abnormal values)
   2. Refer to the action items column for appropriate management.
3. Review client medication lists
   1. Identify indication for each medication on client’s medication list for each cardiovascular risk factor. Discuss with PCP if uncertain of indication.
   2. Review common side effects

## Clinical Considerations Across All Cardiovascular Disease (CVD) Risk Factors

### Dietary and Lifestyle Modifications

Each category corresponds to handouts available for clients.

- Say NO to Sugar Drinks
- Say NO to Junk Food
- Say NO to Processed Foods
- Eat Smart Portions
- Fruits and Vegetables Make Great Snacks!
- Eat Smart Snacks
- Eat Fruit instead of Junk Food
- Eat More Vegetables
- Physical Activity: Move More, Sit Less

ASCVD Risk Estimator from the American College of Cardiology will estimate an individual’s 10-year risk of atherosclerotic cardiovascular disease (ASCVD: acute coronary syndromes, myocardial infarction, stable or unstable angina, arterial revascularization, stroke/transient ischemic attack, peripheral arterial disease). This risk can be reassessed yearly.

- Calculator^2^: <http://tools.acc.org/ASCVD-Risk-Estimator-Plus/#!/calculate/estimate/>

Aspirin use for primary prevention of ASCVD or heart failure and atrial fibrillation^2^

- - Low dose aspirin use (81mg daily) may be considered in adults aged 40-70 at elevated risk, based on ASCVD score and who are NOT at increased bleeding risk.
  - Low dose aspirin should not be given to adults ages >70 years for primary prevention.
  - Low dose aspirin should not be used for primary prevention for adults of any age who are at increased bleeding risk (e.g medication, blood clotting disorders).

Metabolic syndrome is a risk-factor for cardiovascular diseases.

- - Diagnosis: Presence of at least 3 out of 5 risk factors:
    - Elevated waist circumference (≥35 inches for women; ≥40 inches for men)
    - Elevated serum triglycerides (≥150 mg/dl) or receiving drug treatment for elevated triglycerides (e.g. fibrates, nicotinic acid)
    - Reduced HDL cholesterol (<50 mg/dl for women; <40 mg/dl for men) or drug treatment for low HDL cholesterol (e.g. fibrates, nicotinic acid)
    - Elevated blood pressure (≥130/85 mm Hg) or drug treatment for hypertension
    - Elevated fasting glucose (≥100 mg/dl) or drug treatment for diabetes
  - Therapy: Lifestyle therapies are recommended for all persons with metabolic syndrome as well as treatment for individual risk factors.^3^
  - Weight gain is common side effect of many anti-psychotic medications (ie olanzapine).^4^

Smoking cessation – all individuals should be assessed for tobacco smoking and those who smoke should be advised to quit and should be treated with behavioral counseling and pharmacologic treatment.^2,5^

## DIABETES MELLITUS

### Guidelines

|  | **Information** | **Action Item** |
| --- | --- | --- |
| **Who should be screened** | Meets any criteria: ^4,6-8^   - Use of anti-psychotic medications (test yearly and 3,6 months after initiation of anti-psychotic med) - Overweight/obesity (BMI ≥ 25kg/m^2^) with hypertension, history of CVD, first-degree relative with diabetes, HDL <35mg/dl, or triglycerides >250mg/dl (test every 3 years) - Prediabetes (test yearly) - Age ≥45 years (test every 3 years) | - Test using Hemoglobin A1c or fasting glucose. |
| **What is prediabetes?** | Meets any criteria:^9^   - Hemoglobin A1c 5.7-6.4% - Fasting plasma glucose 100-125 mg/dl - Plasma glucose 140-199 mg/dl measured two hours after 75 gram oral glucose tolerance test | - Provide tailored educational handouts on lifestyle modification - Refer to intensive behavioral lifestyle intervention program. - Discuss with PCP about possible use of metformin |
| **Who meets definition of type 2 diabetes mellitus?** | Meets any criteria:^8^   - Hemoglobin A1c ≥ 6.5% - Fasting glucose ≥ 126 mg/dl - Random plasma glucose ≥ 200 mg/dl - Prior diagnosis of type 2 diabetes |  |
| **What is the goal?** | Meet and maintain hemoglobin A1c < 7% to slow progression of complications^10^ |  |
| **What are the treatment options for blood glucose control?** | 1. Dietary and lifestyle modifications^4,11^     AND/OR | - Provide tailored, educational materials with multiple, sessions. Use motivational interviewing techniques. - Refer for medical nutrition therapy and diabetes self-management education with a certified diabetes educator. |
|  | 1. Medications^4,12^ | - Discuss medication with PCP. |
| **How should monitor?** |  | - Obtain A1c every 3 months if A1c >7%. |
|  |  | - Obtain A1c every 6 months if A1c <7%. |
|  |  | - Discuss with PCP about blood sugar monitoring if client takes insulin or has had hypoglycemia. |
| **Who is at risk for hypoglycemia?** | On insulin or sulfonylurea therapy, older age, erratic eating patterns, significant weight loss, and/or exercise^10^ | - Educate client on signs and symptoms |
| **What are the signs of low blood sugar (hypoglycemia)?** | Tremors, palpitations, shakiness, sweating, dizziness, tachycardia, weakness, drowsiness, confusion, seizure, and/or blood sugar ≤70 mg/dl^10^ | - Treat with fast-acting carbohydrates (candy, juice). - If not help, call PCP or send to Emergency Room |
| **What are the signs of high blood sugar (hyperglycemia)?** | Polyuria, polydipsia, weight loss, fatigue, blurred vision, lethargy, trouble concentrating, and/or confusion | - Send to ER if lethargy, confusion, other neurologic symptoms |
| **What other screening and/or treatment is needed?** | Lipid Panel (yearly) | - Discuss with PCP about statin therapy |
|  | Microvascular complications (yearly)^13^ |  |
|  | - Nephropathy (kidney) | - Obtain Urine microalbumin-to-creatinine test |
|  | - Neuropathy | - Obtain Diabetic foot exam |
|  | - Retinopathy/other visual impairment | - Obtain dilated eye exam by Ophthalmology |
|  | Blood pressure control | - See hypertension management. |

### Clinical Considerations for People with Serious Mental Illness

- If individual has higher functional ability, provide additional resources for lifestyle and dietary counseling.
- All patients with type 1 diabetes require insulin^12^. Type 2 diabetes occurs with insulin resistance and relative insulin deficiency^8^. Patients with type 2 diabetes are often prescribed oral diabetes medications^12^.
- Not all patients require daily blood sugar monitoring. However, insulin use requires regular monitoring ^12^. Individuals with SMI and cognitive issues may need oversight and education of eating patterns, medications.
- A history of erratic eating or physical activity patterns or an eating disorder may place an individual at higher risk for side effects or complications from oral diabetes medications or is on insulin.
- Less stringent goal of A1c < 8% may be appropriate for individuals with a history of hypoglycemia, limited life expectancy advanced microvascular or microvascular complications, extensive comorbid conditions.^10^
- If an individual has poorly controlled blood sugars despite interventions, consider referral to Endocrinology.
- Reaching dietary and blood sugar goals may require collaboration and coordination with family members, guardians, residential program staff and/or other social supports.

### Supplemental Medical Information

- Hyperglycemia can lead to dehydration because glucose acts as an osmotic agent.
- Insulin facilitates entry of glucose into cells. When insulin is not present (e.g. insulin not present or insulin-resistant), cells do not receive adequate glucose and they may develop starvation ketosis.
- Metformin reduces hepatic glucose production and improves cellular uptake of glucose and utilization.

Common Medications for Diabetes Mellitus Management**^12^**

| **Drug Class** | **Medication Name** | **Common Doses** | **Major side effects** |
| --- | --- | --- | --- |
| *Biguanides** | Metformin IR (Glucophage) | 500-1000mg twice daily | Diarrhea, nausea, vomiting, infection |
|  | Metformin ER | 500-2000mg once daily |  |
| *DPP-4 inhibitors*  *(“iptins”)* | Sitagliptin (Januvia) | 100mg daily | Headache, dizziness, hypersensitivity reaction, joint pain, pancreatitis |
|  | Saxagliptin (Onglyza) | 2.5-5mg daily |  |
|  | Linagliptin (Trajenta) | 5mg daily |  |
|  | Alopgliptin (Nesina) | 25mg daily |  |
| *GLP-1 receptor agonists***  *(“tides”)* | Liraglutide (Victoza, Saxenda) | 0.6-1.8 mg daily (Victoza)  0.6-3mg daily (Saxenda) | Nausea, vomiting, diarrhea, pancreatitis, hypoglycemia, injection site reaction |
|  | Exenatide (Byetta, Bydureon) | IR: 5-10mcg twice daily  ER: 2mg once weekly |  |
|  | Dulaglutide (Trulicity) | 0.75-1.5mg once weekly |  |
|  | Lixisenatide (Adlyxin) | 10-20mcg daily |  |
|  | Semaglutide (Ozempic) | 7-10mg daily (pill)  0.5-1mg weekly (injection) |  |
| *SGLT-2 inhibitors*  *(“flozins”)* | Canagliflozin (Invokana) | 100-300mg daily | Genitourinary infection, volume depletion, fracture |
|  | Dapagliflozin (Farxiga) | 5-10mg daily |  |
|  | Empagliflozin (Jardiance) | 10-25mg daily |  |
|  | Ertugliflozin (Steglatro) | 5-15mg daily |  |
| *Sulfonylureas* | Glyburide (Diabeta, Glynase) | 2.5-10mg daily | Hypoglycemia, hyponatremia, weight gain, nausea, rash |
|  | Glipizide (Glucotrol) | 2.5-10mg daily |  |
|  | Glimepiride (Amaryl) | 2-4mg daily |  |
| *Thiazolidinediones* | Pioglitazone (Actos) | 15-45mg daily | Hypoglycemia, edema, fracture, heart failure, weight gain |
|  | Rosiglitazone (Avandia) | 4-8mg daily |  |
| *Insulin*** |  |  |  |
| *Rapid-acting* | Lispro (Humalog) | Dose varies by individual | Hypoglycemia, injection site reaction, rash, weight gain, edema |
|  | Aspart (Novolog) |  |  |
| *Regular* | Humalin R, Novalin R |  |  |
| *Intermediate-acting* | NPH (Humalin N, Novalin N) |  |  |
| *Long-acting* | Glargine (Lantus, Basaglar, Toujeo) |  |  |
|  | Detemir (Levemir) |  |  |
|  | Degludec (Tresiba) |  |  |

**First-line therapy*

***Administered subcutaneously (via injection)*

## ELEVATED BLOOD CHOLESTEROL (DYSLIPIDEMIA)

### Guidelines

|  | **Information** | **Action Item** |
| --- | --- | --- |
| **Who would benefit from lipid management?** | Known ASCVD = Secondary prevention for recurrent event^2,3^   - Acute coronary syndrome, myocardial infarction, stable or unstable angina - Ischemic stroke, transient ischemic attack - Peripheral arterial disease   Elevated risk of developing new ASCVD = Primary prevention for new event^2,3^   1. High risk (meets any criteria):  - Adults with diabetes - Age 40-75 years with 10-year ASCVD risk of ≥7.5% and risk enhancers - LDL ≥ 190 mg/dl  1. Moderate risk (meets any criteria):  - Age 20-39 years with family history of premature ASCVD, LDL ≥160mg/dl, or risk enhancers - Age 40-75 years with 10-year ASCVD risk of 5-7.5% and risk enhancers - Age >75 years | - Determine if client has known ASCVD or an elevated risk for new ASCVD - If at risk for new ASCVD, can evaluate for risk enhancers: - Family history of premature ASCVD - Persistently elevated LDL ≥160mg/dl - Chronic kidney disease - Metabolic syndrome - History of pre-eclampsia or premature menopause - Persistently elevated triglycerides ≥175 mg/dl - History of inflammatory disease (e.g. rheumatoid arthritis, HIV) - Ethnicity (e.g South Asian ancestry) |
| **Who should be screened for new diagnosis of dyslipidemia?** | Meets any criteria:^3,4,6^   - Use of anti-psychotic medications^14^ (test yearly if no risk enhancer or every 3,6 months after initiation/dose change). - Age 20-39 years with risk enhancers (frequency guided by clinical factors - Age 40-75 years without diabetes   - No ASCVD risk factors  (test every 4-6 years)   - With ASCVD risk factors  (test yearly) | - Screen with lipid panel - Calculate 10-year (or lifetime) atherosclerotic cardiovascular disease (ASCVD) risk estimation using [ASCVD Risk Calculator](http://tools.acc.org/ASCVD-Risk-Estimator-Plus/%23!/calculate/estimate/) |
| **What is the treatment for dyslipidemia?**  **What is the treatment for dyslipidemia (continued)?** | Lifestyle and dietary modifications^3,4^ (Everyone)  Statin Therapy^3,4^   1. Recommended for all  - Known ASCVD - Diabetes - LDL ≥ 190 mg/dl - ASCVD 10-year risk ≥20% - Ages 40-75 years with ASCVD risk 7.5-19.9% and 1+ risk enhancer  1. May benefit  - High risk and ASCVD 10-year risk 5-7.5% - Age 20-39 years with family history of premature ASCVD, LDL ≥160mg/dl, and/or risk enhancers - Age 40-75 years with ASCVD risk of 5-7.5% and 1+ risk enhancer - Age >75 years | - Provide tailored, educational materials with multiple, educational sessions. Use motivational interviewing techniques. - Recommend high-intensity statin - Discussion with PCP if client would benefit from statin therapy (use ASCVD risk to guide intensity). |
| **How should monitor?** | - Goal to reduce LDL levels by 30-50% based on statin intensity. | - Obtain lipid panel at least once a year - Obtain lipid panel every 1-3 months^3^ if new medication or dose change. - If not at goal, discuss with PCP about adherence, dose, or addition of fenofibrate. |
| **What to do if there are side effects?** | Individuals may benefit from a drug holiday, medication switch, or reduced dosing schedule. | - Call PCP to discuss stopping medication vs dose change and if labs need to be drawn. |
| **What happens if the triglycerides are high (>139 mg/dl) on multiple occasions?** | - Dietary counseling (everyone) - If triglycerides ≥ 500mg/dl consider statin + fenofibrate - If triglycerides 139-499mg/dl and diabetes/risk factors, consider statin + icosapent ethyl (expensive)^15^ | - Provide tailored, educational handouts with multiple, educational sessions. Use motivational interviewing techniques. |

### Clinical Considerations for People with Serious Mental Illness

- If individual has higher functional ability, would provide additional resources for lifestyle and dietary counseling.
- Consumers with SMI likely benefit from screening in absence of traditional CVD risk factors as some anti-psychotic medications have side effects of dyslipidemia.^7,14^
- Discussions with PCP about risks-benefits if individual not strictly meeting clinical criteria.

### Supplemental Medical Information

- A lipid panel includes information on cholesterol and triglycerides. The different types of cholesterol are HDL, LDL, and VLDL. Colloquially, LDL has been known as the “bad cholesterol.” LDL plays a role in the development of atherosclerosis and heart disease.^3^
- Statins inhibit the enzyme (hydoxymethylglutaryl (HMG) CoA reductase), which synthesizes cholesterol.
- Best practice is to use a risk-based approach to guide initiation of statin for individuals without a history of ASCVD, and then to use LDL values to guide intensity of statin therapy and addition of other agents.
- Non-fasting lipid panels are now common practice for most patients and recommended by guidelines.^15^ Fasting lipid panels may be considered with triglycerides are significantly elevated.^3^
- Atypical anti-psychotic medications are associated with raising triglyceride levels.^3,14^ Individuals on these medications likely will benefit from intensive dietary and physical activity modifications for lipid management.
- Ezetimibe is preferred agent in conjunction with statin with primary hypercholesterolemia or insufficient reduction in LDL levels for high-risk individuals.
- Fenofibrate is preferred agent in conjunction with statin to lower triglycerides >500 mg/dl and to reduce likelihood of developing pancreatitis.

### Common Medications for Lipid Management

| **Drug Class** | **Medication Name** | **Common Doses** | **Major side effects** |
| --- | --- | --- | --- |
| *Statins (first line)* |  |  |  |
| Moderate-intensity | Atorvastatin (Lipitor) | 10-20mg daily | Muscle aches or weakness, headaches, nausea, sleep disturbances |
|  | Rosuvastatin (Crestor) | 5-10mg daily |  |
|  | Pravastatin (Pravachol) | 40-80mg daily |  |
|  | Simvastatin (Zocor) | 20-40mg daily |  |
|  | Lovastatin (Altoprev) | 40mg daily |  |
|  | Fluvastatin XL (Lesacol) | 80mg daily |  |
|  | Pitavastatin (Livalo) | 2-4mg daily |  |
| High-intensity | Atorvastatin (Lipitor) | 40-80mg daily |  |
|  | Rosuvastatin (Crestor) | 20-40mg daily |  |
| *Cholesterol absorption inhibitors* | Ezetimibe (Zetia) | 10mg daily | Increase liver enzymes when also taking statin |
| *Fibric acid derivatives** | Fenofibrate (Trillipix, Antara) | Nanocrystal 145mg daily  Micronized 160-200mg daily | Rash, nausea, bloating, cramping, muscle aches. |
|  | Gemfibrozil (Lopid) | 600mg twice daily |  |
| *Bile acid sequestrants (rarely used)* | Cholestyramine (Questran) | 4-25g daily | Nausea, bloating, cramps, constipation, increase in liver enzymes, increase of triglycerides |
|  | Colestipol (Colestid) | 5-30g daily |  |
|  | Colesevelam (Welchol) |  |  |
| *N—3 Fatty Acids** | Iscosapent ethyl | 2g twice daily | Lower extremity swelling, constipation, joint and muscle aches, bleeding, arrythmia |
| *PCSK9 inhibitors* | Alirocumab | 75-150mg every 2 weeks | Injection site reactions |
|  | Evolocumab | 140mg every 2 weeks or 420mg every month |  |

*****Triglyceride-lowering medication

## HYPERTENSION

### Guidelines

|  | **Information** | **Action Items** |
| --- | --- | --- |
| **Who should be screened?** | - Use of anti-psychotic medications (test yearly and 3,6 months after initiation of anti-psychotic med)^4^   OR   - All adults ≥18 years (screen yearly)^16^ |  |
| **How should blood pressure (BP) be measured?** |  | 1. Have individual relax and sit on chair with feet on floor and back supported, for >5 minutes. No talking or cell phone use during measurement. 2. Use an automated BP measurement device that has been validated. Use the correct cuff size. 3. Support the client’s arm (rest on desk) and position middle of cuff on individual’s upper arm. No clothes should be between cuff and individual. 4. Repeat measurement(s) if first reading is ≥120/80 mm Hg. Measure and use average of 2-3 readings^16^ |
| **Who meets definition of hypertension?** | Elevated readings on at least 2 occasions:   - Systolic blood pressure ≥130 mmHg   OR   - Diastolic blood pressure ≥80 mg Hg   OR |  |
|  | On medication for hypertension |  |
| **What is the goal?** | Treatment goal of <130/80 mm Hg^2^ |  |
| **What are the treatment options?** | 1. Lifestyle and dietary modifications^4,16^   AND/OR | - Provide tailored, educational materials with multiple, educational sessions. Use motivational interviewing techniques. |
|  | 1. Medication^4,16^ |  |
|  | - SBP ≥ 130-139 or DBP ≥ 80-89 - SBP ≥ 140 or DBP ≥ 90 - High risk: Prior heart attack, stroke, bypass surgery, stent, diabetes, CKD, ASCVD risk ≥ 10%, age ≥65 years | - Discuss with PCP about blood pressure goals. All high-risk individuals would benefit from strict control with medication(s). |
| **What to do if there are side effects?** |  | - If new lightheadedness, dizzinesss, check blood pressure and call PCP. - If new dry cough, call PCP to discuss if this is a side effect. - If new face, lip, or throat swelling, stop the medication and send to Emergency Room |
| **What if BP is elevated)?** |  | - If BP is > 120/80, ensure repeat blood pressure check every 3-6 months |
|  |  | - If BP is >130/80, discuss with PCP about medication changes; client may need labs. |
| **What if SBP >180 mmg Hg or DBP > 120 mm Hg?** |  | - If client has new acute headache, changes in mental status, signs of a stroke, chest pain, or respiratory distress, send to Emergency room. - If client is asymptomatic, check individual is taking medication as prescribed and discuss with PCP. |

### Clinical Considerations for People with Serious Mental Illness

- If individual has higher functional ability, would provide additional resources for lifestyle and dietary counseling.
- As resources allow, automated blood pressure machines that have been appropriately validated are preferred.^16^
- Current clinical guidelines aim for <130/80 mmHg. Prior guidelines aimed for a target of BP of ≤140/90 mm Hg ^16^. The previous, higher goal may be appropriate for those at high fall risk, with gait instability, age >65 years, difficulties with fluid intake, or a history of electrolyte imbalances (e.g. side effect for medications such as lithium).
- Clinicians may ambulatory blood pressure monitoring to confirm diagnosis and guide treatment.
- Some anti-psychotic medications may lower blood pressure, leading to orthostatic hypotension or impaired blood pressure regulation, by blocking adrenergic receptor.^17,18^
- Clients who present initially with BP ≥140/90 mm Hg may benefit from two-drug therapy.^16^
- Higher rates of adverse effects may occur if consumer is on ACE inhibitor and ARB simultaneously ^16^.
- If poorly controlled blood pressure on multiple agents, consider referral to Cardiology.

### Supplemental Medical Information

- ACE inhibitors and ARBs slow proteinuria; often used in individuals with diabetes.

Common Medications **^16^**

| **Drug Class** | **Medication Name** | **Common Doses** | **Major side effects** |
| --- | --- | --- | --- |
| *ACE Inhibitors** | Lisinopril (Prinivil, Zestril) | 5-40mg daily | Angioedema, Cough, hyperkalemia, hypotension, dizziness, avoid in pregnancy |
|  | Benazepril (Lotensin) | 5-40mg daily |  |
|  | Enalapril (Epaned, Vasotec) | 5-40mg daily |  |
|  | Ramipril (Altace) | 2.5-5mg daily |  |
| *Angiotensin II Receptor Blockers (ARBs)** | Candesartan (Atacand) | 8-32mg daily | Hypotension, dizziness, contraindicated in pregnancy |
|  | Losartan (Cozaar) | 50-100mg daily or twice daily |  |
|  | Irbesartan (Avapro) | 150-300mg daily |  |
|  | Valsartan (Diovan) | 40-320mg daily |  |
| *Beta Blockers* | Atenolol (Tenormin) | 50-200mg daily | Bradycardia, bronchospasm, depression, fatigue, sexual dysfunction, hyperkalemia, worsen heart failure |
|  | Bisoprolol (Zebata) | 2.5-20mg daily |  |
|  | Carvedilol (Coreg) | 3.125-25mg twice daily |  |
|  | Labetalol (Trandate) | 100-400mg two or three times daily |  |
|  | Metoprolol tartrate | 25-100mg twice daily |  |
|  | Metoprolol succinate | 50-400mg daily |  |
|  | Propanolol (Inderal) | 10-80mg two to four times daily |  |
| *Calcium Channel Blockers** | Amlodipine (Norvasc) | 5-10mg daily | Headache, lightheadedness, flushing, lower extremity edema, bradycardia (for diltiazem, verapamil) |
|  | Felodipine | 2.5-10mg daily |  |
|  | Nifedipine ER (Procardia) | 30-90 mg daily |  |
|  | Dilitiazem (Cardizem XT) | 180-360mg daily |  |
|  | Verapamil ER (Calan) | 180-240mg daily or twice daily |  |
| *Thiazide Diuretics** | Hydrochlorothiazide (HCTZ) | 12.5-50mg daily | Electrolyte changes, gout, hyperglycemia, sexual dysfunction |
|  | Chlorthalidone | 12.5-100mg daily |  |
| *Loop Diuretics* | Furosemide (Lasix) | 20-80mg daily | Electrolyte changes, hypersensitivity reaction, ototoxicity |
|  | Torsemide (Demedex) | 5-10mg daily |  |
| *Potassium Sparing Diuretics* | Amiloride | 0.5-2mg daily or twice daily | Gynecomastia, menstrual abnormalities hyperkalemia |
|  | Triamterene (Dyrenium) | 50-100mg daily or twice daily |  |
|  | Eplerenone (Inspra) | 50-100mg daily or twice daily |  |
|  | Spironolactone (Aldactone) | 25-100mg daily |  |
| *Alpha Antagonists* | Doxazosin (Cardura) | 1-16mg daily | Dizziness, fatigue, headache |
|  | Prazosin | 1-10mg two or three times daily |  |

**First line therapy*

# PART II: Cross-Cutting Care Processes to Deliver CVD Risk Factor Care for People with Serious Mental Illness

## POPULATION HEALTH MANAGEMENT

### Overview

*Population health* refers to tracking the overall health of a *group* of individuals (i.e. entire health home population). In this section, we provide guidance on how to use a Population Health Management Database to assist Health Homes programs in delivering targeted cardiovascular care in the community setting.

Population level data is useful for a) systematically identifying and tracking CVD risk factors in your entire population of health home clients, b) determining prevalence of specific CVD risk factors in your health home population (e.g. hypertension, dyslipidemia), and c) tracking the delivery of guideline concordant care.

| **Key Documents and Tools in this Section**   - Population Health Database - Codebook for Database - Lab Ranges   **Other Documents and Tools Referenced in this Section** |
| --- |
| - Clinical Guidelines Bundle (*Clinical Guidelines* section) - Care Plan (*Individual Client Care Plan* section) |

### Identify and Track CVD risk factors

- 1. Fill in ***Population Health Database*** *(if not available in existing electronic health record)*
     1. Use medical record data
     2. Use individual client ***Care Plan***
  2. For each CVD risk factor:
     1. Assess how many clients have the risk factor
     2. Assess how many clients need screening for the risk factor
     3. Assess how many risk factors uncontrolled

### Plan to Improve Specific CVD Risk Factor(s)

- 1. Align the goals and efforts by CUSP team with the needs of the health home population
     1. Choose targets for health home team action
     2. Identify people needing care for a particular issue (using sort function in excel)
  2. Use CUSP as tool to address target.
  3. Use ***Population Health Database*** to track progress of CVD risk factor target in your health home population. The spreadsheet is a summary of individual client changes over time and health home client population changes over time

### Tips for Using the Population Health Database

**Color coding:**

Red = goal not met

Yellow = above but close to goal

None = at goal or not have CVD risk factor

- 1. Enter data for each consumer for each time point. The spreadsheet will automatically color code.
  2. If more than one value exists, such as for blood pressure, use the most recent values.
     1. For 0-months (baseline), enter in most recent data from prior 6 months.
     2. For 6- and 12-months, enter data from the prior interval.
     3. For example, if the study start date was Jan. 1, 2020, then 0-month data should be from July 1, 2019 through December 31, 2019. Then the 6-month data should be from Jan. 1, 2020 through June 30, 2020. And 12-month data should be from July 1, 2020 through Dec. 31, 2020.
  3. Use the clinical guidelines bundle to determine if consumer meets criteria for diagnosis of hypertension, diabetes, and/or high cholesterol
     1. BMI: <https://www.nhlbi.nih.gov/health/educational/lose_wt/BMI/bmicalc.htm>
     2. ASCVD risk score: <http://tools.acc.org/ASCVD-Risk-Estimator-Plus/>
  4. If data is the same as prior time point (e.g. demographics), re-enter the data. All columns need to be filled in for tables to populate accurately.
     1. For yearly labs/exams (eye, foot exam, urine/protein creatinine ratio, lipid labs): If consumer had exam/lab in past year, enter “y” for yes and re-enter data from prior time point if within 1 year.
     2. If the consumer is due (> 1 year), enter “n” for no.
     3. For example, if an eye exam was done on November 15, 2019, this date and “y” would be entered into the baseline 0-month and 6-month data. The consumer would be due again for his/her yearly eye exam on November 15, 2020.
  5. To refresh/update dashboard tables within a single tab
     1. Click on table of interest. Then go to Data (on menu bar) 🡪 Refresh all

OR

- - 1. Save and close spreadsheet. Then reopen spreadsheet.
  1. To filter tables/data: how to select specific rows of data
     1. The first time after data is entered for each sub-group positive for HTN, diabetes, or dyslipidemia, the filters at the top of each table will need to be manually changed to ‘y’ so that only those participants will be displayed in the tables. ***
     2. Click on triangle within column. Select the rows of interest (For example, to see only consumers with data at the 6-month study time point).
     3. To unfilter, click on the triangle again 🡪 Select All to show all data again.

| **Key Categories** | **Contents and Outcomes** |
| --- | --- |
| Data | *Data on individual consumers for each timepoint*  Demographics  Information on each CVD risk factor |
| Summary Tables | *Summary tables of entire health home population at each time point*  Demographics: Age, BMI, Race, Ethnicity, Gender  Received lifestyle information  Diagnosis: Diabetes, Hypertension, Dyslipidemia  Issue and barriers |
| HTN Tables | *Summary tables of consumers diagnosed with hypertension*  Recent BP (past 6 months)  Follow up scheduled in 1 month  Recent BP in past 6 months and poorly controlled (SBP >130 or DBP>80)  Level of blood pressure control with SBP<130 and DBP <80 (strict) or SBP < 140 and DBP <90 (broad)  Average blood pressure and labs (includes: Systolic blood pressure (SBP); Diastolic blood pressure (DBP); A1c; Lipid labs: total cholesterol, HDL, LDL, triglycerides |
| Diabetes Tables | *Summary tables of consumers diagnosed with diabetes*  On Statin  Lipid panel in past 12 months  A1c<7% (well controlled)  A1c in past 6 months  Yearly exams: dilated eye exam, foot exam, urine/protein creatine ratio  Average blood pressure and labs (includes: Systolic blood pressure (SBP); Diastolic blood pressure (DBP); A1c; Lipid labs: total cholesterol, HDL, LDL, triglycerides |
| Dyslipidemia Tables | *Summary tables of consumers diagnosed with elevated cholesterol*  On Statin  Has LDL < 100  Statin recommendation  10-year ASCVD ≥7.5%  With Lipid Panel in past 12 months  Average blood pressure and labs (includes: Systolic blood pressure (SBP); Diastolic blood pressure (DBP); A1c; Lipid labs: total cholesterol, HDL, LDL, triglycerides |

## INDIVIDUAL CLIENT CARE PLAN

### Overview

The *Individual Client Care Plan* details the *individual* client’s health needs, problems and health goals. It includes an individually tailored plan to assist the client in achieving his or her cardiovascular health goals. Developing and maintaining the Individual client care plan will ensure that both client-centered care and guideline-concordant care are implemented.

| **Key Documents and Tools in this Section**   - Care Plan - Educational handouts - Appointment Reminder (for client) - Chronic Disease logs: Blood pressure and blood glucose - Lifestyle Log: Diet and Exercise Tracker - Medication Card for client   **Other Documents and Tools Referenced in this Section** |
| --- |
| - Guides for Effective Communication (*Care Coordination* section) - Population Health Database (*Population Health Management* section) - Clinical Guidelines Bundle (*Clinical Guidelines* section) |

### Create an Individualized *Care Plan*

1. Prior to intake meeting, review records and fill in client’s health needs and goals.
2. Identify hypertension, elevated blood cholesterol, and/or diabetes.
3. Meet with client to identify and assess their needs, goals, and complete the care plan together.

### Use the Care Plan to Guide Actions

1. Identify which CVD risk factors are controlled or poorly controlled. This information should also be recorded in the ***Population Health Database***.
2. If applicable, track blood pressure measurements or blood glucose with ***Chronic Disease logs.***
3. Identify whether client needs updated labs or recently had them done. Use Care coordination tool, ***Guides for Effective Communication***, when reaching out to PCP.
4. Update ***Care Plan*** after meeting with client or clinical update from PCP.
5. Update ***Population Health Database*** (if needed)

### Provide Supportive Self-management

1. Meet with client and identify potential challenges (ie. adherence, dietary challenges)
2. Provide client with self-management tools that they can use. These tools can include ***Chronic Disease Logs***, ***Lifestyle Log, Medication Card.***
3. Provide ***educational handouts*** based on client’s CVD risk factors (refer to ***Clinical Guidelines Bundle***)

## CARE COORDINATION

Overview
In this section, we focus on the Primary Care visit and all related activities before, during and after the visit that involve *Care Coordination* in the Health Home setting. Improving cardiovascular health targets for Health Home clients requires effective communication and coordination between health care providers (i.e. psychiatrist, primary care) and community or social supports (i.e. family, friends, psychiatric rehabilitation program). It also includes ensuring continuity of care, streamlining handoffs to other staff, scheduling appointments, and post-hospital discharge activities.

Care coordination activities are listed in a suggested order of occurrence and reference a set of tools that can help you a) prepare for a visit with PCP, b) gather information and plan during and after a visit with a PCP (or specialist), and c) communicate with other clinical providers outside of regularly scheduled visits.

| **Key Documents and Tools in this Section**   - PCP Phone call log - Medical Release Form – *sites draft their own* - Primary Care Visit Communication Form - Guides for Effective Communication   **Other Documents and Tools Referenced in this Section** |
| --- |
| - Appointment Reminder (*Individual Client Care Plan* section) - Care Plan (*Individual Client Care Plan* section) - Medication Card for client (*Individual Client Care Plan* section) - Clinical Guidelines Bundle (*Clinical Guidelines* section) - Chronic Disease logs: Blood pressure and blood glucose (*Individual Client Care Plan* section) - Population Health Database (*Population Health Management* section) - Educational Handouts (*Individual Client Care Plan* section) - Lifestyle Log: Diet and Exercise Tracker (*Individual Client Care Plan* section) |

### Prior to PCP Visit:

1. Review Health Home client charts for upcoming PCP visit or clients without a PCP visit within one year, or less, if known diabetes, hypertension or high cholesterol.
2. Note upcoming PCP appointment and inform client of appointment using ***Appointment Reminder***. Assist client with making appointments and setting up transportation.
3. Notify staff of upcoming PCP appointments (CUSP team can identify who these people are) via weekly team meetings, or an established appointment tracking and notification system.
4. Update ***Care Plan*** with most recent results of blood pressure(s), blood glucose(s), and labs.
5. Review and update medication list (this will either be the client’s ***Medication Card*** or the ***medication section of the client’s Care Plan)*** and adherence issues. This updated list will accompany the client on their PCP visit.
6. Meet with client prior to PCP visit to prepare for visit.
   1. Client should understand what type (primary care) of visit and what medical issues should be discussed. Consider using ***Guides for Effective Communication – Pre-visit with HH Client.***
   2. Review ***Care Plan*** with client and update sections as needed
   3. Focus on the priority concern (e.g. elevated blood pressure readings, and also refer to the ***Clinical Guidelines Bundle***) and possible outcomes.
   4. Review and update supportive self-management activities using ***Chronic Disease Logs - blood glucose, Chronic Disease Logs - blood pressure, and/or Medication Card***
   5. Complete required ***Medical Release Forms*** (not included in this set of documents), review with client and have client sign.
      1. If HH RN/Staff attending PCP visit with client: Discuss with client that you can help keep track of instructions and changes to their plan of care and communicate health priorities to the PCP. Obtain client’s permission to attend visit.
      2. If Client attending visit on own: Assist client with completion of the ***Primary Care Visit Communication Form*** to take with them to upcoming visit.
7. Send ***Primary Care Visit Communication Form*** ahead of visit to PCP office AND bring a copy in person (if nurse is going to visit).
8. Send ***Appointment Reminder*** to client the day before visit, confirm transportation and appointment details.

### During PCP Visit:

1. Attend PCP visit with client. Bring the following documentation: ***Primary Care Visit Communication Form, Chronic Disease Logs (blood glucose, blood pressure), Medication Card, Medical Release Form***
2. Address poorly controlled cardiovascular disease risk factors, known barriers and other guideline-concordant care concerns w/PCP in-person.
   1. Use a patient centered approach, and Motivational Interviewing principles to guide discussion. For examples, see ***Guides for Effective Communication***
   2. Encourage action-oriented clinical decision-making. Or examples, see ***Guides for Effective Communication – During visit with PCP – Example of Conversation Flow***
3. Fill in ***Primary Care Visit Communication Form*** based on PCP visit instructions or orders.
4. Obtain prescription and/or lab orders (if indicated).
5. Schedule follow up visit before leaving office.
6. Obtain copies of the visit summary.

### After/in between PCP visit:

1. Update Health Homes ***Care Plan*** with changes from the visit: blood pressure, labs, med changes, follow up visit date and time, issues to follow up on (e.g. check blood glucose log, take interim BP measures, ensure that labs ordered are obtained).
2. Enter information into the ***Population Health Database***.
3. Meet with client to follow up on PCP visit and confirm understanding in areas identified.
4. Hand-off to staff/PCP (verbal or written).
5. Facilitate client obtaining labs, imaging tests, specialist consultations, etc. (scheduling and logistics).
6. Report blood pressure results, medication adherence issues back to PCP as requested.
7. Request medication changes, lab orders or results, visit summary or other information from the PCP. For examples, see ***Guides for Effective Communication - Between visits with PCP*** and record using ***PCP Phone call log***
8. Address needs in the following areas as they arise:

- *Labs* – ensure that orders for blood work and other diagnostics are obtained.
- *Follow-up visits* – schedule the next appointment, use the ***Appointment Reminder*** form to remind the client of the appointment.
- *Pharmacy* – if a new prescription or change in prescription was ordered, ensure that it is picked up from the pharmacy, and the client’s medication list (***Medication Card*** and ***medication section of the client’s Care Plan)*** is updated accordingly.
- *Insurance* – troubleshoot problems with insurance coverage and renewal for clients as needed.
- *Transportation* – if transportation is required, notify the transportation coordinator.
- *Housing* – know what staff person to refer client to for residential/housing concerns.
- *Family supports* – know when family members or other social supports can be engaged to assist client with meeting health goals.
- *Supportive Self-Management* – provide individualized teaching using the ***Educational Handouts, Lifestyle Log-Diet and Exercise Tracker*** as needed***.***

### Post-Hospital Discharge

1. Track Hospitalizations (medical, psychiatric)
2. Obtain discharge summary
   1. Identify changes to prior care plan (e.g. medication changes, appointments)
   2. Identify action items and/or new health issues
3. Communicate and clarify changes with healthcare providers. Action items may include:
   1. Additional testing (e.g. imaging, laboratory blood draws)
   2. Follow-up appointments
   3. Medications (new medications, change of dose, or stopping prior medication)
4. Work with client and healthcare providers to complete testing and follow-up appointments.

## Additional Tools/Handouts

### Educational handouts

**Say NO to Sugar Drinks**

**What is a Sugar Drink?** A **Sugar Drink** is any drink that has sugar and calories.

Avoiding sugar drinks can help you lose weight and be healthier as you manage your blood pressure, diabetes and cholesterol and/or weight.


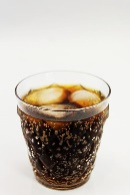

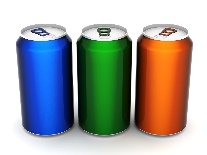
Say **NO** to:

1. **Regular Soda**


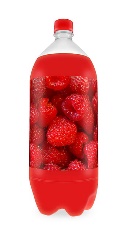


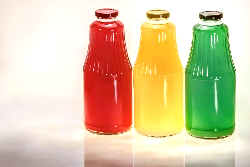


1.
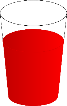
**Fruit Punch**


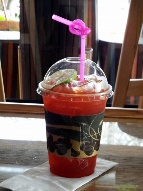


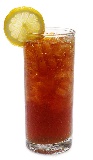


1. **Sweet Tea**


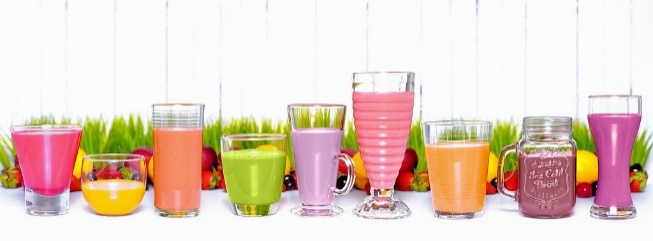

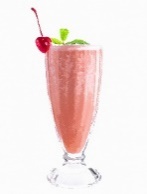


1. **Milkshakes/Smoothies**


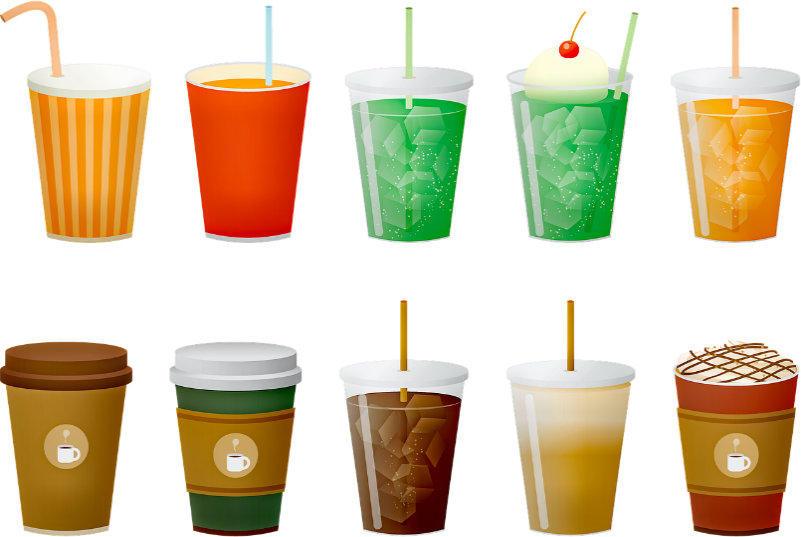

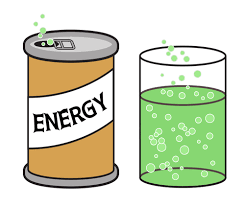


1. **Sports/Energy Drinks**
2. **Fancy Coffee Drinks**

**Eat Fruit Instead of Junk Food**

Fruit is a healthier option for when you want something sweet to eat. What counts as a serving of fruit^*^?

-
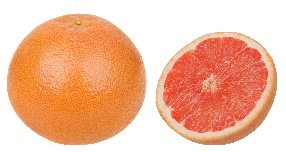
1 whole piece of fruit such as a medium apple, banana or orange
- half of a grapefruit


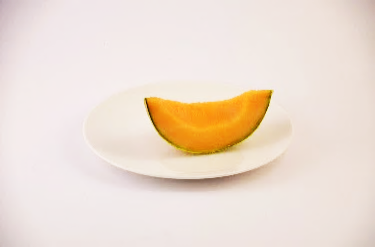


- a melon wedge


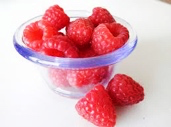


-
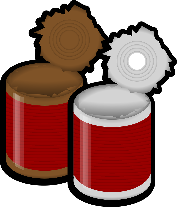
½ cup of berries


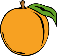


-
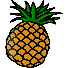
½ cup of cooked or canned fruit


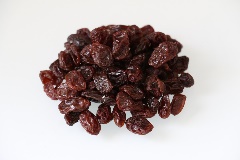


- ¼ cup of dried fruit like raisins or prunes


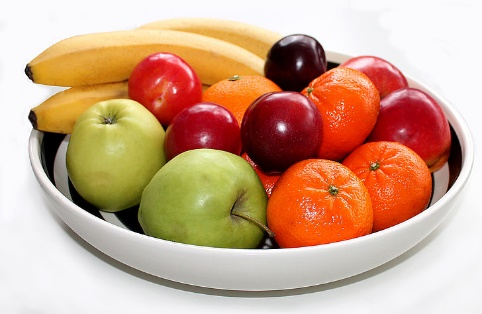


*Fruit juice is also a way to add fruit into your diet but best to avoid because it contains a lot of sugars.

**Fruits and Vegetables Make Great Snacks!**

Fruits and vegetables contain vitamins, minerals and fiber. Eating fruits and vegetables instead of unhealthy foods can help you manage your blood pressure, diabetes, cholesterol and/or weight.


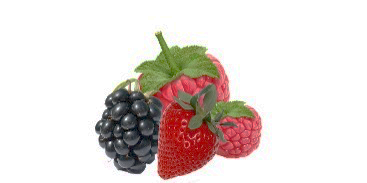

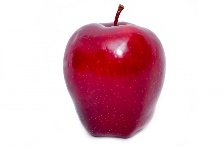


Apple Berries


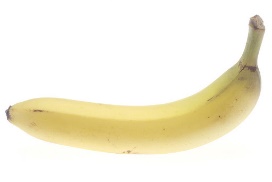

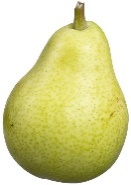


Banana Pear


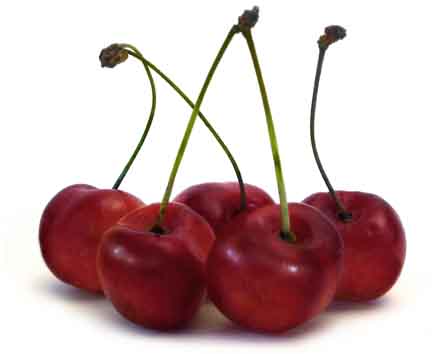

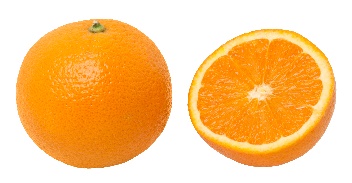


Orange Cherries


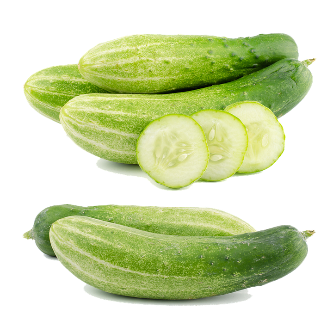

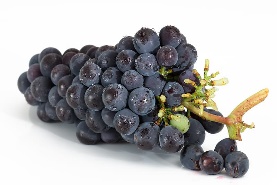


Grapes Cucumber


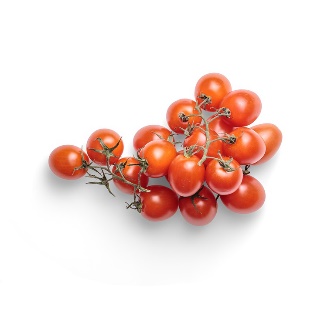


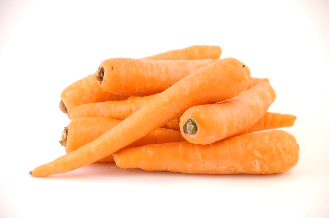


Cherry Tomatoes Carrots


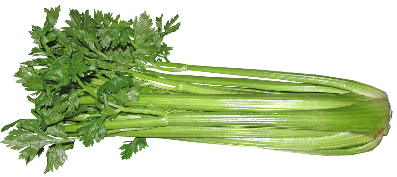

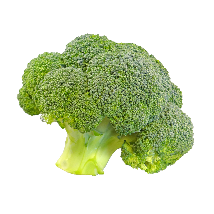


Broccoli Celery

**Say NO to Junk Food**

Junk foods are foods that are sugary sweet, salty or greasy. They are high in calories and/or fat, and have little to no nutritional value. Eating junk food can cause you to gain weight and can have a negative effect on your blood pressure, diabetes, and cholesterol.

Try to say NO to these types of food:

-
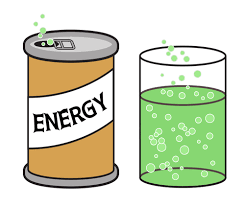

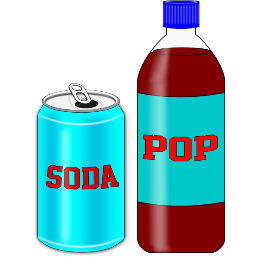
Sweets


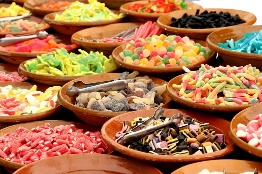


- - Sugary Drinks
  -
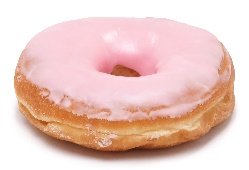
Candies
  - Baked goods and pastries


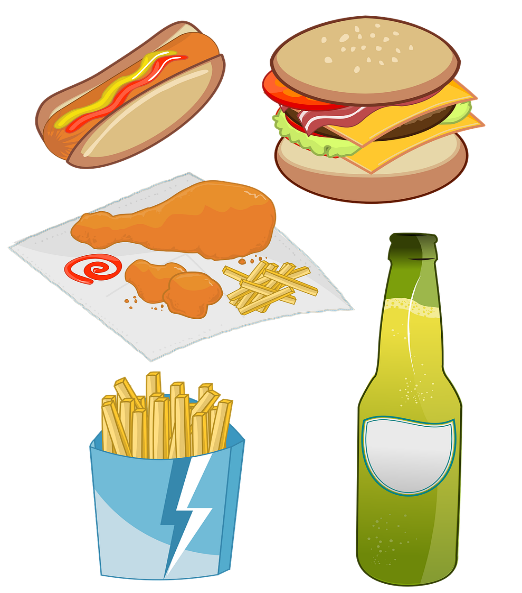

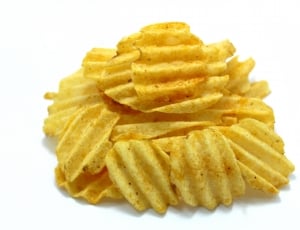


- Salty/Greasy/Fried Foods
  - Chips
-
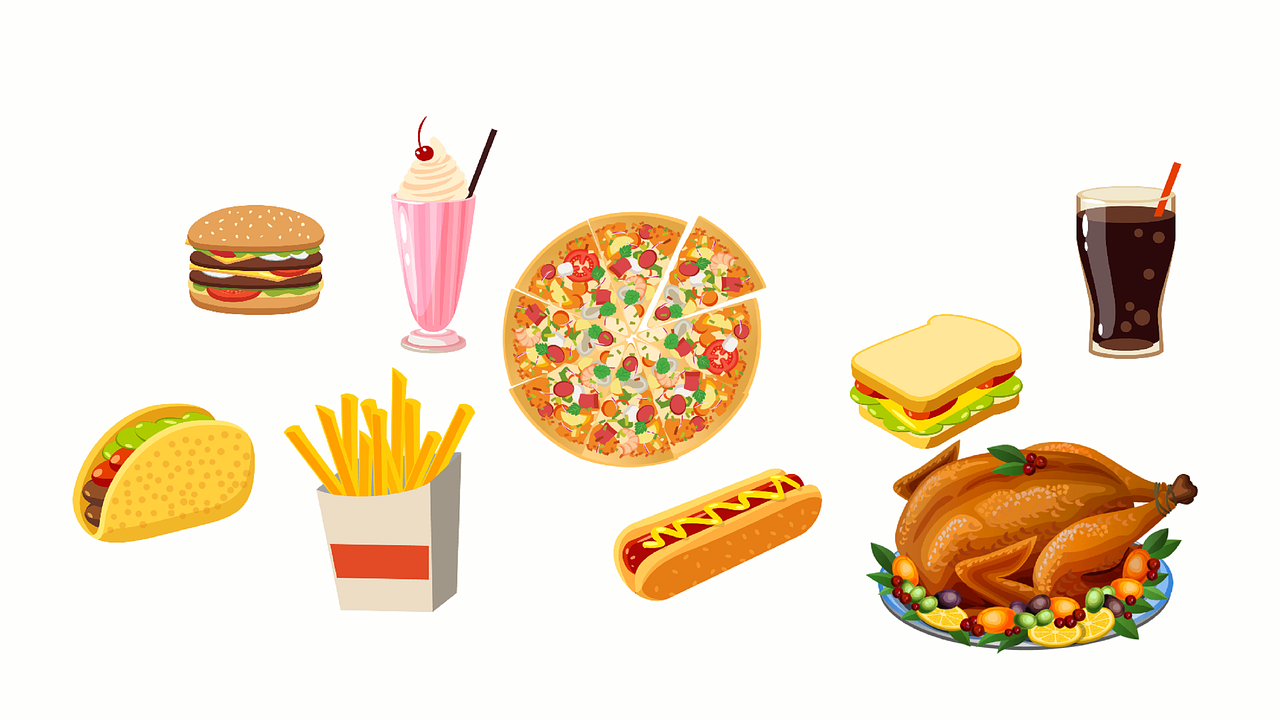

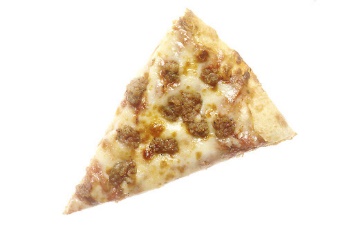
French Fries
- Pizza
- Fast Food

**Eat More Vegetables**

Vegetables are an important part of a healthy diet. They include nutrients and fiber and can help you manage your blood pressure, diabetes, cholesterol and weight. Try adding some of these vegetables to your next meal! Consider beans and peas as a great source of low fat protein.


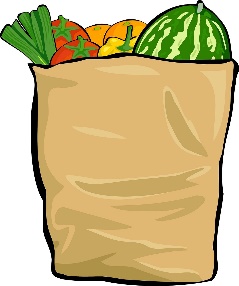


| **DARK GREEN VEGETABLES**   Broccoli   Turnip Greens   Collard Greens   Dark Green Lettuce   Spinach   Kale   Mustard Greens   Romaine Lettuce    **STARCHY VEGETABLES**   Corn   Green peas   Green Lima Beans   Potatoes    **RED & ORANGE VEGETABLES**   Carrots   Pumpkin   Red Peppers   Sweet Potatoes   Tomatoes |  | **OTHER VEGETABLES**   Asparagus   Beets   Brussels Sprouts   Cabbage   Cauliflower   Celery   Cucumbers   Green Beans   Green Peppers   Mushrooms   Onions   Zucchini  **BEANS & PEAS**   Black Beans   Black-Eyed Peas   Kidney Beans   Lentils   Chickpeas   Navy Beans   Pinto Beans   Soy Beans   White Beans |
| --- | --- | --- |

**Say NO to Processed Foods**

Processed foods are foods that mostly come in boxes, bags, or cans*. These foods are high in calories, fat, and sodium, and have low nutritional value. They fill your body with things it doesn’t need.

To help control your blood pressure, diabetes, cholesterol and/or weight, try to avoid processed foods.

Say NO to processed foods:


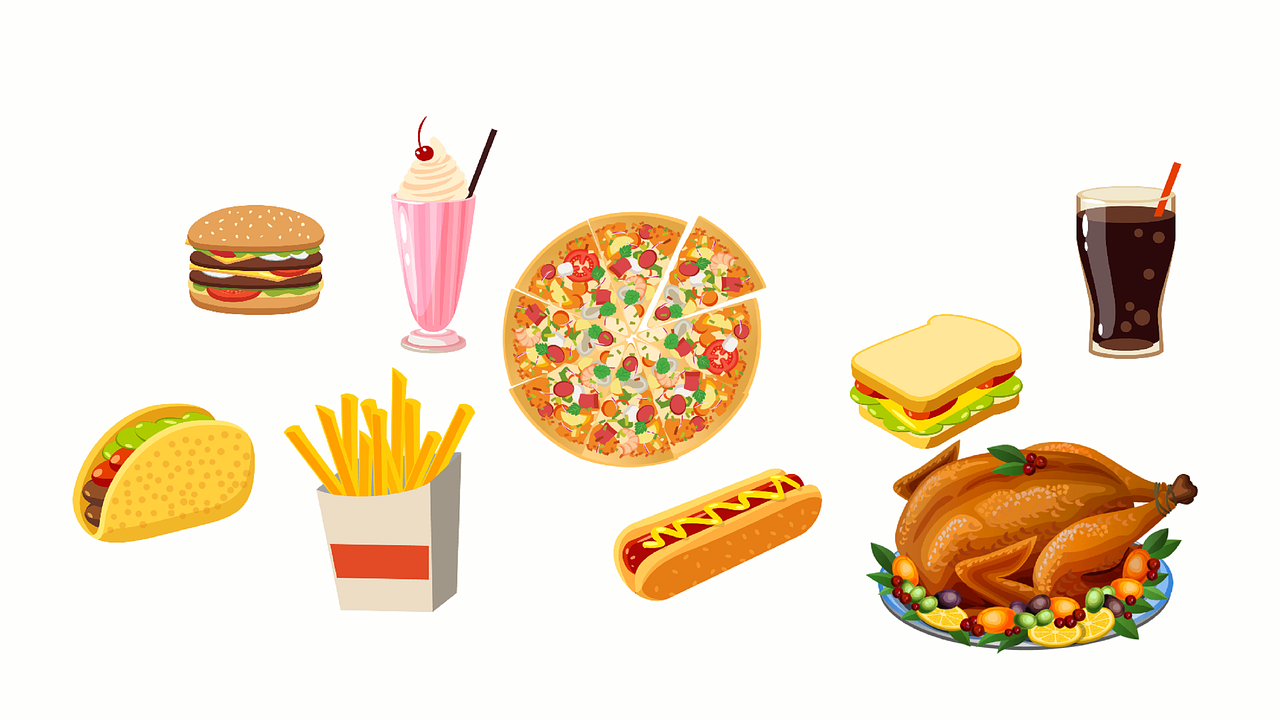


|  |  |
| --- | --- |

*Canned fruit in its own juice and No-added-salt canned veggies are okay to eat!

**Eat Smart Portions**

A **Smart Portion** is one serving as defined by the Nutrition Facts label. Eating less than you would normally eat is also a way to eat smarter portions.

Eating smart portions can help you reduce the number of calories you are consuming and help you manage your blood pressure, diabetes, cholesterol and weight.

Have a food label? Look for the serving size at the top of the Nutrition Facts label.

Don’t have a food label? No problem! Your hand can help you figure out smart portion!


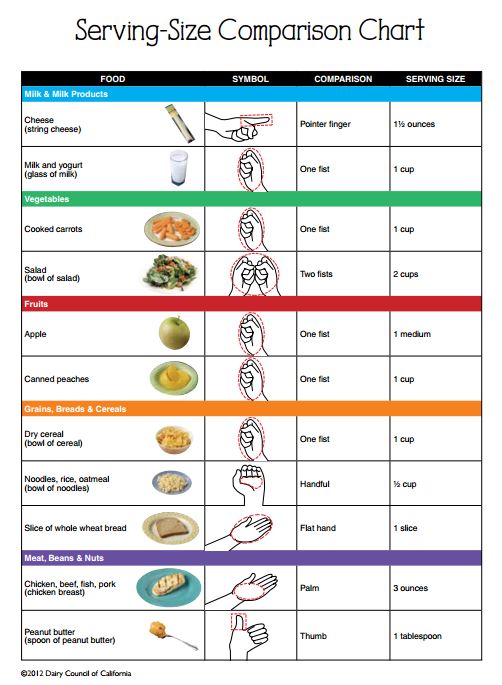

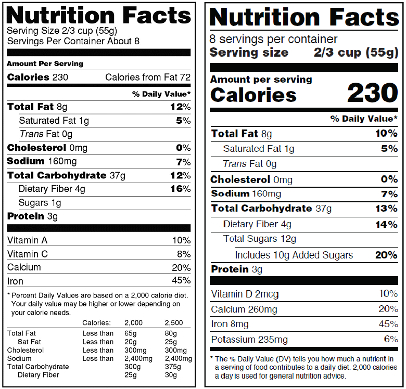


**Eat Smart Snacks**

A **Smart Snack** is a snack that is eaten when you are physically hungry *AND* is a healthy, low-calorie food. Aim for snacks that are 200 calories or less.

Eating smart snacks can help you manage your blood pressure, diabetes, cholesterol and weight.


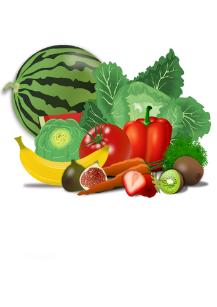
Examples of Smart Snacks include:

- Fruits and Vegetables


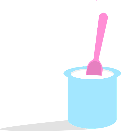


- Yogurt
-
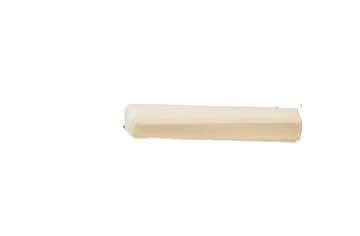
Low-fat string cheese


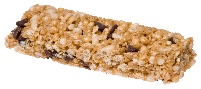


-
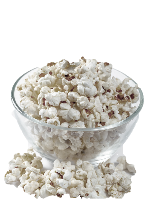
Low-calorie granola bar
- Popcorn (no butter)


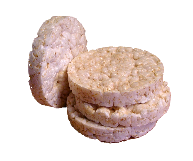


- Rice cakes


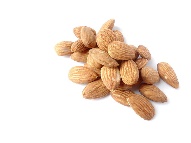


- 1/3 cup of nuts


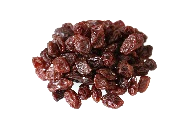


- ¼ cup of raisins

**Physical Activity: Move More, Sit Less!**

Being active can help manage your blood pressure, diabetes, cholesterol, and weight.

The recommended amount of physical activity needed to be healthy is 150 minutes per week of moderate intensity aerobic activity. This can be broken down into:

- 30 minutes on 5 days/week
- 20 minutes on 7 days/week

New to being active? That’s OK! You can build up to 150 minutes per week. You can work up to the recommended amount by increasing the time as you get stronger.

Remember, any amount of moving is better than none. Start by sitting less, taking the stairs instead of the elevator, and going for 10 minutes walks.

Other examples of moderate-intensity aerobic activity include:

Brisk walking

Bicycling


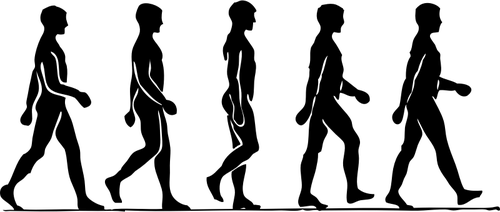
Swimming laps

Mowing the lawn (push mower)

Gardening

Dancing

### Appointment Reminder

**APPOINTMENT REMINDER**

Health Home Client/Patient Name _______________________________ DOB ________________

Contact # for appointment reminder: ( ) ____________________

You have an appointment with your (please check):

❒ **Primary Care Clinician**

❒ Specialist (cardiologist, endocrinologist, diabetes educator)

❒ Other: _____________________________________

❒ Psychiatric provider

❒ Therapist

Appointment information:

Doctor’s Name

Address

Number

On (Date) ________________/_____/______ at ______________ a.m./p.m. (circle)

Month day year time

Person taking you to the appointment: ___________________________________

Transportation: ❒ mobility ❒ cab ❒ staff ❒ family ❒ other

----------------------------------------------------------

--------------------------------------------------------------------------------

**PLEASE BRING THE FOLLOWING TO YOUR APPOINTMENT:**

For Health Home Nurse accompanying client to appointment

- PCP Communication Form (Health Home Nurse/Staff)
- HEALTH HOME CLIENT CARE PLAN – *review & update prior to appointment*

Or

For client attending on own or with PRP/RRP staff

- PCP Communication Form (For Health Home patient/or support person)/HEART HEALTH REPORT CARD *- Fill in Heart Healthy Report Card section for patient going on own.*
- List of all your medications
- Blood pressure or blood sugar diary (Chronic Disease logs if you are tracking

### Chronic Disease Logs:

#### Blood Glucose Log

My Goal Blood Sugar is: _________________ **Call my doctor if my Blood Sugar is > _____< ______**

| **Day of Week** | **Date** | **Breakfast** | | **Lunch** | | **Dinner** | | **Nighttime** | | **Comments:** |
| --- | --- | --- | --- | --- | --- | --- | --- | --- | --- | --- |
|  |  | Before | Post | Before | Post | Before | Post | Before | Post |  |
| Sunday | ____/___/____ |  |  |  |  |  |  |  |  |  |
| Monday | ____/___/____ |  |  |  |  |  |  |  |  |  |
| Tuesday | ____/___/____ |  |  |  |  |  |  |  |  |  |
| Wednesday | ____/___/____ |  |  |  |  |  |  |  |  |  |
| Thursday | ____/___/____ |  |  |  |  |  |  |  |  |  |
| Friday | ____/___/____ |  |  |  |  |  |  |  |  |  |
| Saturday | ____/___/____ |  |  |  |  |  |  |  |  |  |
| Sunday | ____/___/____ |  |  |  |  |  |  |  |  |  |
| Monday | ____/___/____ |  |  |  |  |  |  |  |  |  |
| Tuesday | ____/___/____ |  |  |  |  |  |  |  |  |  |
| Wednesday | ____/___/____ |  |  |  |  |  |  |  |  |  |
| Thursday | ____/___/____ |  |  |  |  |  |  |  |  |  |
| Friday | ____/___/____ |  |  |  |  |  |  |  |  |  |
| Saturday | ____/___/____ |  |  |  |  |  |  |  |  |  |

####
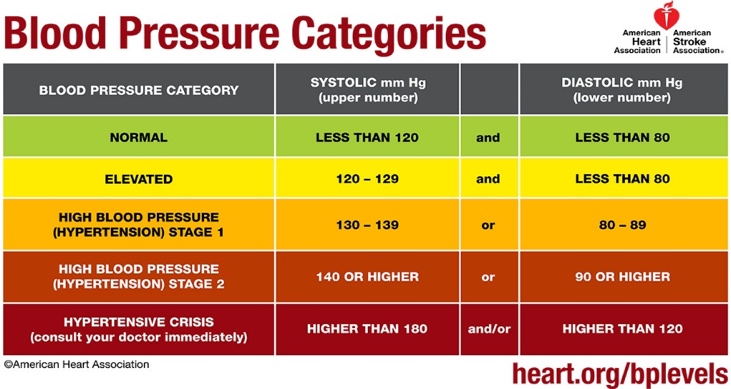
Blood Pressure Log

**BLOOD PRESSURE TRACKER**

For Home Monitoring

Please bring this with you to your doctor’s appointment.

**Name: __________________________________**

**Goal Blood Pressure: _________/ ___________**

**Call physician if above: _________/ ___________**

| Date | Time  (Circle AM or PM) | Systolic/Diastolic | Heart Rate (pulse) | Comments  *(example: feeling stressed or angry, just exercised, missed BP medication)* |
| --- | --- | --- | --- | --- |
|  | AM/PM | / |  |  |
|  | AM/PM | / |  |  |
|  | AM/PM | / |  |  |
|  | AM/PM | / |  |  |
|  | AM/PM | / |  |  |
|  | AM/PM | / |  |  |
|  | AM/PM | / |  |  |
|  | AM/PM | / |  |  |
|  | AM/PM | / |  |  |
|  | AM/PM | / |  |  |
|  | AM/PM | / |  |  |
|  | AM/PM | / |  |  |
|  | AM/PM | / |  |  |
|  | AM/PM | / |  |  |
|  | AM/PM | / |  |  |
|  | AM/PM | / |  |  |
|  | AM/PM | / |  |  |
|  | AM/PM | / |  |  |

**How to use a home blood pressure monitor**

• Be still. Don't smoke, drink caffeinated beverages or exercise within 30 minutes before measuring your blood pressure. Empty your bladder and ensure at least 5 minutes of quiet rest before measurements.

• Sit correctly. Sit with your back straight and supported (on a dining chair, rather than a sofa). Your feet should be flat on the floor and your legs should not be crossed. Your arm should be supported on a flat surface (such as a table) with the upper arm at heart level. Make sure the bottom of the cuff is placed directly above the bend of the elbow. Check your monitor's instructions for an illustration or have your healthcare provider show you how.

•Measure at the same time every day, especially after a change in treatment and during the week before your next appointment.

•Take multiple readings and record the results. Each time you measure, take two or three readings one minute apart and record the results using a tracker.

•Don't take the measurement over clothes.

**Choosing a home blood pressure monitor**

The American Heart Association recommends an automatic, cuff-style, bicep (upper-arm) monitor.

•Wrist and finger monitors are not recommended because they yield less reliable readings.

•Choose a monitor that has been validated. If you are unsure, ask your doctor or pharmacist for advice.

•When selecting a blood pressure monitor for a senior, pregnant woman or child, make sure it is validated for these conditions.

•Make sure the cuff fits — measure around your upper arm and choose a monitor that comes with the correct size cuff.

**Once you’ve purchased your monitor, bring it to your next appointment**

Have your doctor check to see that you are using it correctly and getting the same results as the equipment in the office. Plan to bring your monitor in once a year to make sure the readings are accurate.

### Lifestyle Log: Diet and Exercise Tracker

Name: ___________________________________ Week of: ________________________ Weight:___________________lbs.

**Lifestyle Log - Diet and Exercise Tracker**

| **DAY** | **MON** | **TUE** | **WED** | **THU** | **FRI** | **SAT** | **SUN** |
| --- | --- | --- | --- | --- | --- | --- | --- |
| **Did you AVOID:** | | | | | | | |
| **Sugar Drinks** | **Yes No** | **Yes No** | **Yes No** | **Yes No** | **Yes No** | **Yes No** | **Yes No** |
| **Salty/Greasy Foods** | **Yes No** | **Yes No** | **Yes No** | **Yes No** | **Yes No** | **Yes No** | **Yes No** |
| **Sweets** | **Yes No** | **Yes No** | **Yes No** | **Yes No** | **Yes No** | **Yes No** | **Yes No** |
| **Processed Foods** | **Yes No** | **Yes No** | **Yes No** | **Yes No** | **Yes No** | **Yes No** | **Yes No** |
| **Did you EAT:** | | | | | | | |
| **5 Fruits & Veggies** | **1 2 3**  **4 5** | **1 2 3**  **4 5** | **1 2 3**  **4 5** | **1 2 3**  **4 5** | **1 2 3**  **4 5** | **1 2 3**  **4 5** | **1 2 3**  **4 5** |
| **Smart Portions** | **Yes No** | **Yes No** | **Yes No** | **Yes No** | **Yes No** | **Yes No** | **Yes No** |
| **Did you EXERCISE?** | | | | | | | |
|  | **Yes No** | **Yes No** | **Yes No** | **Yes No** | **Yes No** | **Yes No** | **Yes No** |

### Medication Card for client

**Medication Card**

| **Patient Name:**  **Medication List as of (date):** | | | | | | |
| --- | --- | --- | --- | --- | --- | --- |
| **Name** | **Used For** | **Instructions** | **Morning**   | **Afternoon**   | **Evening**   | **Night**   |
|  |  |  |  |  |  |  |
|  |  |  |  |  |  |  |
|  |  |  |  |  |  |  |
|  |  |  |  |  |  |  |

Page _____/_____

### Primary Care Physician Phone Call Log

| Date & Time of call | HH Client | PCP contacted & # | Reason for call | Result |
| --- | --- | --- | --- | --- |
|  |  |  | - Date & Time of next appt - Schedule next Appt. - Labs - After-visit Summary - Alert PCP - Report follow-up result - Request med change - Other:____________________ | - Next appt - Labs Rec’d - Visit Summary Rec’d - PCP aware - Med change - Other: |
|  |  |  | - Date & Time of next appt - Schedule next Appt. - Labs - After-visit Summary - Alert PCP - Report follow-up result - Request med change - Other:____________________ | - Next appt - Labs Rec’d - Visit Summary Rec’d - PCP aware - Med change - Other: |
|  |  |  | - Date & Time of next appt - Schedule next Appt. - Labs - After-visit Summary - Alert PCP - Report follow-up result - Request med change - Other:____________________ | - Next appt - Labs Rec’d - Visit Summary Rec’d - PCP aware - Med change - Other: |
|  |  |  | - Date & Time of next appt - Schedule next Appt. - Labs - After-visit Summary - Alert PCP - Report follow-up result - Request med change - Other:____________________ | - Next appt - Labs Rec’d - Visit Summary Rec’d - PCP aware - Med change - Other: |

### Primary Care Visit Communication Form

**Health Home Client:___________________________________________ DOB: __________________**

**PCP: _________________________ PCP Address:___________________________________________**

**Date and Time of Appointment: ___________________________________________**

❒ Person accompanying client to appointment:___________________________ ❒ Client alone

**Remember to bring to your appointment!** ❒ List of medications ❒Blood pressure/ blood sugar diary

**Cardiovascular Risk Factor Priorities**

❒ **Hypertension** (systolic and diastolic BP =/> 130/80 mmHg)* ❒ Controlled ❒ **Uncontrolled**

BP ______/______, Date:________ BP ______/______, Date:_________

Complete this section PRIOR to PCP visit

❒ **Diabetes type 1 or 2** (HgA1C > 6.4%) ❒ Controlled ❒ **Uncontrolled**

HgA1C _______% Date of result: ________________

❒ **Dyslipidemia** (Total Cholesterol > 200 mg/dL, LDL > 130 mg/dL) ❒ Controlled ❒ **Uncontrolled**

ASCVD Score **______%,** Total Chol ______, LDL-C ______, HDL-C ______, Tri ______

Date of result:___________________

❒ Other Risk Factor Information: ________________________________________________________

Complete this section DURING the PCP visit

| ❒ Lifestyle counseling   - Diet and physical activity - **Smoking cessation** | ❒ Referral to specialist (type) |
| --- | --- |
| ❒ Labs ordered   - HgA1C - Fasting lipid panel - Other ____________________ | ❒ Follow up in _________days/weeks/months  On ____/____/_____ at _____­____a.m./p.m. |
| ❒ Medication change(s)  Medication:__________________  Dose:_________Frequency: ______  Indication: ____________________ | ❒ Other issues  ________________________________  ❒ Health Home Nurse needs to know:  ________________________________ |

**What happened at this visit:** *your doctor can help fill this part out*

For Care Coordination Nurse use only:

❒ No changes this visit ❒ Reassess in _____days/weeks/months

❒ Guidelines given to PCP ❒ BP measures/glucose log provided ❒ labs requested

### Guides for Effective Communication

**Overview**

The purpose of this document is to share helpful communication strategies and sample language to promote guideline-concordant care for patients’ hypertension, diabetes, and elevated cholesterol levels. The information and sample scripts provided are to be used when preparing for and/or attending primary care provider (PCP) appointments. Sample language for between visit conversations is also included. This document may be used as a reference for Health Home (HH) nurses, trained lay support staff, and/or family members. It provides guidance for communication with patients and clinicians to encourage action-oriented clinical decision-making and to improve cardiovascular disease risk factor control. For situations where providers are reluctant to adhere to guideline concordant care, there are also suggestions for dealing with such barriers.

**General Strategies**

**Guidelines for effective communication:**

- Use clear and concise language.
- Use a patient-centered approach.
- Ask client how they prefer to be addressed.
- Draw on current clinical guidelines and the client’s specific history to focus discussion.
- Work with initial suggestions from PCP and discuss how best to communicate updates on progress.
- Request clear time line (dates and times) for follow-up on issues.
- Identify the ways that the health home can support the client’s goals to self-manage disease and adhere to medications.

**Pre-medical visit with health home client**

*Hi, _____________, thank you for meeting with me today. You are enrolled in the Health Homes program. We provide you support for your physical health in addition to your mental health. You have a medical doctor’s appointment coming up and this is an opportunity to talk to your doctor about your (blood pressure, diabetes, etc.). Sometimes patients find it helpful when someone else is there to help them talk to their doctor and keep track of what the doctor says or recommends; would it be ok if I or your service coordinator/residential counselor were to attend that visit with you for that purpose?*

If client says, “no”:

Acknowledge their refusal and gently try to determine what factors may be contributing to their hesitation.

Ask: *What are your concerns about this upcoming visit? Tell me a little more about your hesitation. We see this visit as an opportunity to get your major health concerns addressed since it may be many more weeks or months that you are seen again.*  *How would you prefer to communicate your concerns to your doctor at this next visit? How can I help you prepare to discuss this on your own with your doctor?*

If client says, “yes”:

*Thank you! I have been keeping track of your blood pressure and noticed that it is higher than it should be. We want the top number to be lower than 130 and the bottom number to be lower than 80. Your upcoming medical appointment is an opportunity to discuss a plan for getting your blood pressure under control. Let’s list the things that you’ve been working on to do that (make list together). It’s important that we share that information with your doctor. He or she might want to change how much medication you are taking to control your blood pressure. Sometimes, doing those lifestyle things you talked about aren’t enough on their own to keep your blood pressure at a healthy level and medication is the most effective option. What thoughts do you have about that? Any concerns with us bringing any of this up at the visit?*

**During Visit with PCP**

- General guide for conversation with PCP
- Examples of conversations focusing on specific cardiovascular risk factors (hypertension, diabetes mellitus, high cholesterol) follow this section

1. Introduction – introduce self and role in patient’s health

*Hi, my name is ________________. Your patient is enrolled in the Medicaid Health Homes program. This is a program aimed at improving coordination between physical health and behavioral health providers. The goal being improving health outcomes for our clients where Medicaid reimburses mental health programs for a nurse and staff to assist in improving physical health. One area that we have been focused on is cardiovascular-related health. Here is a copy of the letter we faxed to you earlier describing the program in more detail.*

1. Encourage client/patient to speak for self, but if patient is unwilling or unable, with patient’s permission, gently step in to provide further detail of priority concerns for this visit.
2. Provide measures of risk factor control (e.g. blood pressure log, glucose log)
3. Detail lifestyle and self-management strategies that the patient has been following and any adherence issues. Include any other modifiable or non-modifiable risk factors that may contribute to the clinical picture beyond the results provided or seen at the visit.
4. Ask for PCP’s recommendations for management and timeline for follow up. Refer to the clinical bundle for optimal management parameters and timeline for follow up (e.g. follow up in 3 months for an A1C of 9%)
5. Discuss with physician that Health Homes staff will provide follow up blood pressure measures, labs as appropriate, ongoing lifestyle counseling and support, medication monitoring for compliance and effectiveness, as well as assist with continuity of care among psychiatric rehabilitation program, residential rehabilitation program, health homes, etc. team members.
6. Acknowledge client’s commitment to improving their health and thank physician for their support.

*Thank you for your time, Dr. __________! I am encouraged that _______ is seeing you about (insert CVD risk factor) and that we have an opportunity to close the loop between your recommendations and outside of this office visit. I look forward to following up with you!*

Clients with multiple risk factors:

Clients may have more than one cardiovascular risk factor that may need to be addressed over time. There are many reasons why a PCP may not be able to address all risk factors at every visit or may be reluctant to make more than one medication change at a time. Therefore, if the client is in need of a medication adjustment for more than one risk factor, separate, sequential visits may be required.

Regardless, sample language about how to bring up the multiple topics is listed below.

One exception to addressing multiple risk factors may be around laboratory testing. For example, if a client has diabetes and high cholesterol, then it may be appropriate to have an HgbA1C and fasting lipid panel drawn at one time.

**Hypertension**

- Example script is provided in italics.
- Definition: Blood pressure >130/80 mm Hg or on medication for high blood pressure.

*Today we would like to address _________’s blood pressure with you. We have been measuring their blood pressure at our program and are concerned that it’s still elevated (show blood pressure log). We measured these blood pressures at the mental health program with X (automated if applicable) blood pressure machine. _______________ has been working on [list behavioral goals around (*be specific to patient*) cutting out added salt, and losing weight], but we’re not sure that lifestyle modifications alone will be enough to improve their blood pressure targets. What would you consider an appropriate blood pressure target for _______________? What time frame would you recommend for re-evaluating their blood pressure?*

| If PCP measures blood pressure and it is <130/80 mm/Hg: | If client is not on any medication for blood pressure: | If client is taking blood pressure medication but not controlled: |
| --- | --- | --- |
| *I see that it’s better today, but would it be ok if we continue to monitor it and fax you the results if they remain high for us? Could we plan for a blood pressure follow up appointment in two weeks?* | *___________ is not currently on any medication for their blood pressure and we wanted to know what your recommendations are for starting a first-line anti-hypertensive at this point?* | *__________ is on __________(*medication[s]*) for their blood pressure and taking it as directed, but it doesn’t seem to be keeping their pressure at target. What might you consider for better control?* |
|  |  |  |
| If PCP intensifies blood pressure medication*:* | If PCP suggests solely lifestyle modifications: | If PCP does not agree with lower BP targets for patients*:* |
| *That’s great! I will check in with _________’s coordinators and residential staff to ensure that they receive this new [dose of] medication and we will continue to monitor their blood pressure at the program. In addition, we will keep working on the behavioral goals that we mentioned earlier. When would you like to see ____________ next for a follow up?* | *_________ has identified these lifestyle modifications to work on; _____________. I’m concerned that _____ has worked on this in the past but the blood pressure remains above goal. What is your timeline for trying lifestyle modifications before initiating medications? We are happy to continue to check ______’s blood pressure in our health home. Should we send you these readings after a certain period of time? When should he/she schedule the next appointment?* | *We understand that the goals for blood pressure are now recommended to be lower than what was used in the past. What are your concerns with lowering blood pressure to 120’s/70’s for ______? _________has a family history of hypertension, a BMI of ____, and limited resources for healthy food choices and exercise opportunities, so I’m concerned that they may not be able to rely on lifestyle modifications alone for reducing their blood pressure.* |

**Diabetes**

- Example script is provided in italics.
- Definition: HgbA1C >7% or on medication for glucose control

*Today we would like to check in on ________’s diabetes. Their most recent fasting HgA1C on __/___/___ was >6.5% (and if applicable, fasting plasma glucose >125 mg/dL). I see that today’s point of care glucose is ___________. What are your goals for optimal glucose control for ______________? We understand that lifestyle modifications; following a low glycemic diet, losing weight, increasing physical activity are all key to improving blood glucose control, but we’re concerned that this may not be enough. [List lifestyle, dietary modifications that client has been working on]. In addition, __________’s anti-psychotic medication can also increase blood sugars. What would be your next steps for improving blood glucose control at this point?*

| If participant self-monitors the blood glucose and is on medication for diabetes: | If the PCP takes a wait and watch it approach: | If the A1C is VERY uncontrolled and lifestyle modifications aren’t effective: |
| --- | --- | --- |
| *Here are the M/______’s home blood sugars values that were taken _____ (list time of day and how long it was after eating). They are taking (list medications). I have created a medication instruction log for ________ to make it easier to keep track of taking their diabetes medications and monitoring the daily blood glucose levels. In addition to diet modifications, do you have any recommendations for what would bring the morning BG down further? Would they benefit from medication intensification? What is your schedule for checking in on A1C again?* | *What is your timeline for following up on lifestyle recommendations before making changes to their medications? Should we plan to check the A1c in 3 or 6 months?* | *What are your goals for glycemic control? If medication intensification and lifestyle modifications aren’t working at this point, what would be the next step? Would a trial of continuous glucose monitor be appropriate at some point?* |

**High Cholesterol Example Script**

- Example script is provided in italics.
- Definition: ASCVD score >7.5%, presence of diabetes, or LDL>190 mg/dl

*Today we would like to check in on their cholesterol. We reviewed ___________’s most recent lipid results on _____, and calculated their 10-year ASCVD risk score. We noticed that ________‘s LDL remains > 130 mg/dL and/or their ASCVD risk score is > 9%, what are your goals for treating elevated LDL-C? Do you use the ASCVD calculator to guide treatment decisions when a patient’s LDL is not elevated but they have multiple risk factors for developing atherosclerotic disease? __________’s anti-psychotic medication can also increase cholesterol.*

| If client has elevated LDL and high cholesterol and PCP is hesitant to starting statin: | If client has diabetes and PCP has not discussed statin therapy, then advocate for statin initiation: |
| --- | --- |
| *__________ has worked on lowering their cholesterol by eating out less and choosing healthier snacks, and after several months the LDL is still elevated, when would you consider it appropriate to start a statin? How soon after starting would you expect to see an improvement in the LDL? We will continue to work with _________ on lifestyle modifications but when can we check in on the lipids again to see if there’s been an improvement?* | *_________has diabetes and is taking medications for diabetes, but isn’t on a statin currently, the ACC and ADA guidelines indicate that it might be appropriate to start patients with diabetes on a low dose statin. Do you think _______ is a good candidate for a statin? If not, what are your concerns?* |

**Between visits with PCP**

The Health Homes Nurse, PRP or RRP staff may need to initiate a phone call to a client’s primary care provider (PCP) office for any number of reasons including:

- Determine the next date and time of an appointment (for priority cardiovascular concern).
- Schedule an appointment; either an annual exam if none in the last year, the next follow-up appointment (to reevaluate a high blood pressure reading, or elevated HgbA1C, or lipid panel), or a missed appointment.
- Obtain a copy of lab reports; current HgbA1C and/or lipid panel results, or any other tests that are required prior to initiating/intensifying a cardiovascular medication (e.g. liver and kidney function tests, basic metabolic panel)
- Request an after-visit summary; when a client has been seen recently by their PCP (for office blood pressure, current medication list, new prescriptions, and plan of care).
- Alert the PCP to a concerning value (e.g. elevated blood pressure, elevated or low blood sugar) or medication question (e.g. non-adherence) obtained by the Health Home Nurse at the program.
- Report follow-up blood pressure measurement(s) as requested by the PCP.
- Follow up on labs ordered pending medication initiation or change as determined in the last visit’s plan of care.

**Example script of phone call to PCP in between visits is provided in italics.**

*Hi, my name is _________________. I’m assisting Dr. ______ ‘s (insert PCP name) patient, M/________________ with their medical care as part of the Medicaid Health Homes program. Can you please tell me when their next appointment with __________________is scheduled? I would like to schedule their next appointment with __________________. When is your next available appointment?*

Obtaining orders for labs or scheduling appointment

*The Medicaid Health Homes nurse is following M/_________________’s cardiovascular risk factors and would like a copy of their most recent A1C and lipid result. Whom should I reach out to obtain those? If they need to be ordered, do I need to schedule an appointment for M/_______________with Dr. _____________in order to get those updated?*

Request summary of previous visit

*M/____________________ participates in the Medicaid Health Homes program and receives care coordination assistance for their medical needs. They were seen in your office last month, and I would like to request a copy of that the after-visit summary.*

*Update on self-management values*

*When Dr. _____________ last saw M/______________ for their (CVD risk factor), they asked that we obtain _______ measurements at the program and report them back. What is the best way to get that information to Dr. ___________? How can I expect to be notified should a follow up appointment based on these measure.*

## References

1. Cohen J. *Statistical Power Analysis for the Behavioral Sciences*. 2nd ed. ed. Lawrence Erlbaum Associates; 1988.

2. Arnett DK, Blumenthal RS, Albert MA, et al. 2019 ACC/AHA Guideline on the Primary Prevention of Cardiovascular Disease: Executive Summary. *Circulation*. Mar 17 2019:Cir0000000000000677. doi:10.1161/cir.0000000000000677

3. Grundy SM, Stone NJ, Bailey AL, et al. 2018 AHA/ACC/AACVPR/AAPA/ABC/ACPM/ADA/AGS/APhA/ASPC/NLA/PCNA Guideline on the Management of Blood Cholesterol. *Circulation*. Nov 10 2018:Cir0000000000000625. doi:10.1161/cir.0000000000000625

4. Firth J, Siddiqi N, Koyanagi A, et al. The Lancet Psychiatry Commission: a blueprint for protecting physical health in people with mental illness. *The lancet Psychiatry*. Jul 8 2019;doi:10.1016/S2215-0366(19)30132-4

5. Cather C, Pachas GN, Cieslak KM, Evins AE. Achieving Smoking Cessation in Individuals with Schizophrenia: Special Considerations. *CNS drugs*. Jun 2017;31(6):471-481. doi:10.1007/s40263-017-0438-8

6. Consensus development conference on antipsychotic drugs and obesity and diabetes. *The Journal of clinical psychiatry*. Feb 2004;65(2):267-72.

7. De Hert M, Detraux J, van Winkel R, Yu W, Correll CU. Metabolic and cardiovascular adverse effects associated with antipsychotic drugs. *Nature reviews Endocrinology*. Oct 18 2011;8(2):114-26. doi:10.1038/nrendo.2011.156

8. 2. Classification and Diagnosis of Diabetes: Standards of Medical Care in Diabetes-2019. *Diabetes care*. Jan 2019;42(Suppl 1):S13-s28. doi:10.2337/dc19-S002

9. 3. Prevention or Delay of Type 2 Diabetes: Standards of Medical Care in Diabetes-2019. *Diabetes care*. Jan 2019;42(Suppl 1):S29-s33. doi:10.2337/dc19-S003

10. 6. Glycemic Targets: Standards of Medical Care in Diabetes-2019. *Diabetes care*. Jan 2019;42(Suppl 1):S61-s70. doi:10.2337/dc19-S006

11. 5. Lifestyle Management: Standards of Medical Care in Diabetes-2019. *Diabetes care*. Jan 2019;42(Suppl 1):S46-s60. doi:10.2337/dc19-S005

12. 9. Pharmacologic Approaches to Glycemic Treatment: Standards of Medical Care in Diabetes-2019. *Diabetes care*. Jan 2019;42(Suppl 1):S90-s102. doi:10.2337/dc19-S009

13. 11. Microvascular Complications and Foot Care: Standards of Medical Care in Diabetes-2019. *Diabetes care*. Jan 2019;42(Suppl 1):S124-s138. doi:10.2337/dc19-S011

14. Marder SR, Cannon TD. Schizophrenia. *N Engl J Med*. Oct 31 2019;381(18):1753-1761. doi:10.1056/NEJMra1808803

15. Michos ED, McEvoy JW, Blumenthal RS. Lipid Management for the Prevention of Atherosclerotic Cardiovascular Disease. *N Engl J Med*. Oct 17 2019;381(16):1557-1567. doi:10.1056/NEJMra1806939

16. Whelton PK, Carey RM, Aronow WS, et al. 2017 ACC/AHA/AAPA/ABC/ACPM/AGS/APhA/ASH/ASPC/NMA/PCNA Guideline for the Prevention, Detection, Evaluation, and Management of High Blood Pressure in Adults: A Report of the American College of Cardiology/American Heart Association Task Force on Clinical Practice Guidelines. *Journal of the American College of Cardiology*. May 15 2018;71(19):e127-e248. doi:10.1016/j.jacc.2017.11.006

17. Polcwiartek C, Kragholm K, Schjerning O, Graff C, Nielsen J. Cardiovascular safety of antipsychotics: a clinical overview. *Expert Opinion on Drug Safety*. 2016/05/03 2016;15(5):679-688. doi:10.1517/14740338.2016.1161021

18. Leung JY, Barr AM, Procyshyn RM, Honer WG, Pang CC. Cardiovascular side-effects of antipsychotic drugs: the role of the autonomic nervous system. *Pharmacology & therapeutics*. Aug 2012;135(2):113-22. doi:10.1016/j.pharmthera.2012.04.003
